# Sporulation at reduced water activity impairs germination kinetics of *Bacillus subtilis* spores

**DOI:** 10.1128/aem.00677-25

**Published:** 2025-06-10

**Authors:** Víctor Freire, Santiago Condón, Elisa Gayán

**Affiliations:** 1 Department of Animal Production and Food Science, AgrFood Institute of Aragon (IA2), Faculty of Veterinary, University of Zaragoza-CITA 535556, Zaragoza, Spain; Centers for Disease Control and Prevention, Atlanta, Georgia, USA

**Keywords:** *Bacillus subtilis*, spores, sporulation, water activity, salinity, germination, solute, coat, permeability

## Abstract

While the effect of sporulation temperature on spore germination has been extensively studied, the effect of other relevant environmental factors such as water activity (*a_w_*) has been overlooked, despite the fact that sporulation niches with lower humidity than ideal culture media, such as soil, are common in nature. In this work, we characterized the germination kinetics of *B. subtilis* 168 spores produced at reduced *a_w_* (0.98) using either glycerol (S_gly_ spores) or NaCl (S_salt_ spores) in comparison to spores prepared under optimal conditions (S_control_ spores, *a_w_* ~0.99) in a variety of nutrient and chemical stimuli, along with the effect of thermal activation. Spores produced at reduced *a_w_* showed impaired germination to varying degrees depending on the nutrient and solute used to depress *a_w_*. While S_gly_ spores exhibited germination defects in all the nutrients tested (a rich growth medium, L-alanine, L-valine, and the AGFK mixture) compared to S_control_ spores, S_salt_ populations showed an impaired response to L-alanine and L-valine. These nutrient germination defects of the S_gly_ and S_salt_ spores could not be reversed by heat activation. In addition, both populations displayed impaired germination in Ca-DPA, but increased germination rate in dodecylamine. The phenotypes of spores produced at reduced *a_w_* suggested plausible alterations in their coat properties. Using mutant spores with coat morphogenesis defects and a 4 kDa FITC-dextran probe permeability test, we could infer that the impaired germination of S_salt_ spores in nutrients and Ca-DPA may involve alterations in the crust and/or outer coat, leading to reduced permeability.

**IMPORTANCE:**

Bacterial spores are causative agents of relevant zoonoses and foodborne diseases and are involved in food spoilage. Natural sporulation niches, such as soil, are exposed to a variety of changing environmental conditions, such as temperature and water activity (*a_w_*), which strongly influence the dynamics of spore germination. This work provides the first data on the effect of lowering the *a_w_* of the sporulation medium on spore germination kinetics, which, together with previous data on the effect of other environmental sporulation conditions and inter- and intraspecific variability, will help accurately predict germination and thus prevent the negative impacts of pathogenic and food spoiling spores. Furthermore, we inferred that alterations related to the coat are associated with impaired nutrient germination of spores produced at reduced *a_w_* with the addition of NaCl. These findings may help develop novel and efficient strategies to control germination or eradicate spores.

## INTRODUCTION

Sporulation is one of the most enduring survival strategies developed by certain members of the phylum Firmicutes, leading to the formation of dormant spores capable of withstanding external stresses for extended periods while spreading widely in natural resources. Toxigenic spore-formers are causative agents of human and animal diseases, including foodborne illnesses, a risk that is becoming increasingly severe due to climate change (1, 2). In addition, *Bacillus* and *Clostridium* spp. are major contributors to food spoilage (3), which results not only in loss of nutrient resources but also in significant economic and environmental impacts (4). On the other hand, spore resistance and dormancy properties are attracting remarkable interest for biotechnological applications. Spores serve as growth promoters and biological weapons against plant diseases, probiotics, and platforms for the expression of recombinant proteins that can be used for biosensing, bioremediation, cancer therapy, and healing of materials (5–7).

Dormant spores do not pose a threat or exert their beneficial effects until they encounter germination-inducing molecules, such as amino acids, sugars, minerals, or a combination of these. These compounds penetrate through the coat and cortex of spores to reach the specific germinant receptors (GR) located in the inner membrane (8, 9). The interaction between germinants and GR initiates the germination process, leading to the release of dipicolinic acid chelated in a 1:1 ratio with Ca^2+^ (Ca-DPA) in the spore core, enzymatic hydrolysis of the cortex, water influx into the core, and subsequent resumption of metabolism as an active vegetative cell (10).

It is well known that spores exhibit large heterogeneity in germination when exposed to a germinant: not all spores initiate germination at the same time; some tend to do so extremely slowly or remain unresponsive, and the duration of each germination phase varies from spore to spore (11, 12). Such heterogeneity results from intrinsic variation among isogenic spores (12, 13), as well as from variation in ecophysiological properties among species and strains (14). In addition, extrinsic factors acting throughout the life cycle of spore-formers, such as environmental conditions during sporulation and exposure of spores to damaging agents, can modulate spore germination (15, 16). Accurate prediction of spore germination kinetics, taking into account all possible sources of variability, is essential to prevent the negative impacts of pathogenic and food spoiling spores, as well as to promote the full exploitation of spore benefits.

Among the environmental sporulation conditions, the effect of sporulation temperature on germination kinetics has been extensively studied. The influence of this factor depends on the strain, the type and concentration of the germinant, the physicochemical properties of the germination medium, and the previous exposure to a heat activation treatment (15, 17, 18). However, less attention has been paid to the influence of other relevant environmental sporulation conditions, such as water activity (*a_w_*). Nguyen Thi Minh et al. (19) reported that *B. subtilis* ATCC 31324 spores produced at *a_w_* 0.95 depressed with either glycerol or NaCl were able to outgrow at a lower *a_w_* threshold compared to spores produced under optimal conditions (*a_w_* ~0.99). However, the kinetics of spore germination were not addressed. It is of growing importance to address this knowledge gap as soil is a common sporulation niche in nature (1), and soil salinization is expected to increase with climate change (20, 21), potentially making spores produced under suboptimal *a_w_* more abundant.

The objective of this work was to characterize the germination kinetics of *Bacillus subtilis* 168 spores produced at reduced *a_w_* (0.98) using different solutes (glycerol or NaCl) in comparison to spores prepared under optimal conditions (*a_w_* ~0.99). The research encompassed an examination of spore responses to various nutrient and chemical stimuli, along with the impact of thermal activation. In addition, we explored the mechanistic explanation for the nutrient- and Ca-DPA-induced germination impairment observed in spores produced at high salinity.

## MATERIALS AND METHODS

### Strain construction

The *B. subtilis* 168 strain, kindly provided by Prof. R. Kolter (Harvard University), and its deletion mutants Δ*cotY::erm*(Erm^R^) and Δ*cotE::erm*(Erm^R^), referred to in this work as Δ*cotY* and Δ*cotE*, respectively, were used throughout this investigation. The deletion strains were constructed by SPP1 phage transduction as previously described (22), using as donor strains the deletion mutants (BKE11750 and BKE17030) from the BKE genome-scale deletion library (NBRP - National BIO-Resource Project, Japan) developed by Koo et al. (23). Briefly, 0.2 mL of a culture of the donor strain in TY broth (LB [Oxoid, Basingstoke, England] supplemented with 10 mM MgSO_4_ [Panreac, Barcelona, Spain] and 100  µM MnSO_4_ [Carlo Erba, Barcelona, Spain]) was infected with 0.1 mL serial dilutions of an SPP1 phage stock provided by the Bacillus Genetic Stock Center (Columbus, OH, USA) for 15 min at 37°C. A 3 mL volume of TY soft agar (TY supplemented with 0.5% wt/vol of bacteriological agar [Oxoid]) was added to each sample and poured onto TY plates (TY supplemented with 1.5% wt/vol of bacteriological agar). After overnight incubation at 37°C, the plates with abundant plaques were harvested by scraping and vortexing. The supernatant was filtered through a 0.45 µm syringe membrane and stored at 4°C until use. For transduction, recipient cells were grown at 37°C in 2  mL of TY broth until the early stationary phase was reached. Subsequently, 1-, 10-, or 100 µL aliquots of the suspension containing the SPP1 donor phage were added to 1 mL of the receptor cell culture. Immediately after, 9  mL of TY broth was added to each sample, and the transduction mixture was incubated at room temperature for 30 min. Cells were then recovered on TY plates supplemented with erythromycin (1 µg/mL; Sigma-Aldrich, St. Louis, MO, USA) and lincomycin (12.5 µg/mL; Sigma-Aldrich) to select for transduced clones. The replacement of the target gene was initially scrutinized by PCR with primer pairs attaching outside of the deleted region (Table S1) and further verified by sequencing. Afterward, the strains were stored at −80°C in nutrient broth No. 2 (NB; Oxoid, Basingstoke, UK) supplemented with 25% glycerol (Panreac).

### Obtention and purification of spore suspensions

A single colony streaked on nutrient agar (Oxoid) supplemented with 0.6% yeast extract (Oxoid) (NAYE) was inoculated into a 60 mL flask containing 10 mL of NB and incubated overnight at 37°C with shaking (130 rpm; Heidolph Promax 1020, Schwabach, Germany). Afterward, a volume of 20 µL from the culture was inoculated into 250 mL flasks containing 20 mL of liquid 2 × SG sporulation medium (24). The *a_w_* of the sporulation medium was reduced to 0.98 by the addition of 2.75% (wt/vol) NaCl (Panreac) or 8.5% (wt/vol) glycerol, leading to S_salt_ and S_gly_ populations, respectively. Importantly, these conditions produced an equal degree of stress in terms of sporulation efficiency (24). The *a_w_* was measured at room temperature using a Decagon CX-1 (Decagon Devices Inc., Pullman, WA, USA). Simultaneously, a sporulation batch in which no additional solutes were added was included as a control (S_control_ population). To ensure equal maturation time among sporulation conditions, flasks were incubated at 37°C with shaking (130 rpm; Heidolph Promax 1020) until two equal spore counts, determined by plating aliquots previously exposed to a thermal treatment (75°C, 15 min), were obtained on two consecutive days (24): 4 and 6 days when reducing *a_w_* with salt or glycerol, respectively, and 4 days for the control samples. Spores were harvested by centrifugation at 3,345 *g* for 20 min at 4°C and washed three times with distilled water. Spores were then purified by buoyant density centrifugation using Nycodenz, as previously described (13, 24). Spore purity (98% bright spores) was verified by phase-contrast microscopy (Nikon Eclipse E400, Tokyo, Japan), and the suspensions were stored at −20°C until use. To assess the biological variability, three different spore populations were obtained on independent working days at each condition.

### Nutrient-induced germination assays

Nutrient-induced germination assays were performed in NB supplemented with 0.6% (wt/vol) yeast extract (NBYE)—as an example of a complex germinant matrix—or in 25 mM HEPES buffer (pH 7.0) with a saturating concentration (10 mM) of L-alanine (Sigma-Aldrich), L-valine (Sigma-Aldrich), or the mixture AGFK (L-asparagine [Amresco Inc., Solon, Ohio, USA], D-glucose [Panreac], D-fructose [Panreac], and KCl [Panreac]) (25–27). NBYE was supplemented with 4 mg/mL of ampicillin sodium salt (Sigma-Aldrich) to avoid the interference of the growth of the first germinated spores (28). The presence of the antibiotic did not affect the germination behaviour (data not shown). Spores were suspended in each germination medium at a final optical density at 600 nm (OD_600_) of 0.5 (± 0.1). When indicated, spores were heat-activated for 30 min at temperatures between 55°C and 85°C in a PCR machine (T100 Thermocycler, Bio-Rad, Hercules, CA, USA) and then cooled down to 4°C prior to nutrient exposure. Germination kinetics were monitored by OD_600_ in a multiwell plate reader (CLARIOstar Plus, BMG, Ortenberg, Germany), which automatically measured values every 3 min for 4 h while shaking 30 s between readings to avoid spore sedimentation. Germination curves were constructed using the percentage of OD_600_ fall (OD_t_ / OD_i_ × 100, where OD_i_ and OD_t_ represent the initial value and the value measured at subsequent incubation times, respectively). When indicated, germination kinetics were also followed by DPA-Tb fluorescence, with each sample supplemented with a final concentration of 50 µM TbCl_3_ (Sigma-Aldrich). Fluorescence was measured automatically with the above-mentioned multiwell plate reader using similar kinetic parameters and an excitation and emission wavelength of 270 nm and 545 nm, respectively (26). For each sample, the fluorescence at different time points (F_t_) was normalized to the fluorescence of an autoclaved sample (F_i_). The autoclaved sample represents the maximum estimated fluorescence value, corresponding to the total release of DPA and, therefore, complete germination of the population. Background fluorescence from a blank sample containing only the germination medium and TbCl_3_ (F_B_) was subtracted from both F_t_ and F_i_. Germination curves were then constructed by plotting the percentage of DPA release over time, calculated as [(F_t_ − F_B_) / (F_i_ − F_B_)] ×100, for each population. All nutrient germination assays were carried out at 37°C.

After each spectrophotometric or DPA-Tb fluorometric assay, the percentage of germinated spores was determined by phase-contrast microscopy. A total of 100 to 150 individuals per sample were examined and counted as either dormant (phase-bright) or germinated (phase-dark and phase-gray) spores. For each nutrient and environmental condition, we obtained three germination curves from different biological replicates.

### Chemical-induced germination assays

For Ca-DPA-induced germination, spores (OD_600_ 0.5 ± 0.1) were incubated in 25 mM HEPES buffer of pH 7.0 with DPA (Sigma-Aldrich) and CaCl_2_ (VWR, Radnor, Pennsylvania, USA), both added to a final concentration of 50 mM. For dodecylamine-induced germination, spores (OD_600_ 0.5 ± 0.1) were incubated in 25 mM Tris-HCl buffer of pH 9.0 (Sigma-Aldrich) supplemented with 250 mM NaCl, 5% DMSO (Sigma-Aldrich), and 1 mM dodecylamine (Sigma-Aldrich). Germination kinetics in Ca-DPA were assessed by spectrophotometry at 37°C (29), while germination kinetics in dodecylamine were monitored by DPA-Tb fluorescence at 45°C (30), as described above. The percentage of germinated spores at the end of the Ca-DPA germination assays was examined by phase-contrast microscopy. For each chemical, we obtained three germination curves from different biological replicates.

### Modeling of germination curves

Germination curves obtained from OD_600_ measurements were fit to the One-Phase Decay equation (Eq. 1) using GraphPad PRISM 5.0 (GraphPad Software Inc., San Diego, CA, USA). This model describes germination curves using two parameters: the germination rate constant *k* (min^−1^), which represents the speed at which spores germinate during spectrophotometric assays, and the *plateau*, which indicates the percentage of OD_600_ fall at infinite time. When a delay phase was evident prior to the exponential decay segment, theequation 2 was used instead: this model incorporates the *delay* parameter to indicate the duration of such a phase. To evaluate the goodness of fit, the R^2^ and root mean square error (RMSE) were calculated. Curves in which the final OD_600_ decrease was less than 15% were not modeled.


(Eq. 1) $${OD}_{600}\text{ fall }(\%)=(100-plateau)e^{\left(-kt\right)}+plateau$$

(Eq. 2) $${OD}_{600}\text{ fall }(\%)=(100-plateau)e^{\left(-k(t-delay)\right)}+plateau$$

For a more accurate comparison of germination efficiency at the end of the assays, the study used microscopic observation rather than the percentage of OD_600_ decrease at the plateau phase due to different light absorption properties of coat-deficient spores (31).

### Hydrophobicity assays

The hydrophobicity associated with the polysaccharides of the outermost layers of spores was determined by the Bacterial Adhesion To Hydrocarbon (BATH) test, as described by Faille et al. (32) with some modifications. Briefly, a 700 µL spore sample with an OD_600_ of 1.0 was centrifuged, resuspended in McIlvaine buffer of pH 7.0 (33), and incubated at 37°C for approximately 20 min without shaking. Subsequently, a 300 µL aliquot of the spore suspension was mixed with 100 µL of hexadecane (Sigma-Aldrich) in an Eppendorf tube, vortexed for 3 min, and allowed to stand at 37°C for 30 min to enable phase separation. Afterward, 125 µL of the aqueous phase was carefully extracted and transferred to a 96-well plate to measure OD_600_ (OD_w+h_). For comparison, the OD_600_ was measured on an additional sample that was treated in the same manner but without mixing with hexadecane (OD_w_). Surface hydrophobicity was expressed as the percentage of OD_600_ remaining in the aqueous phase in the sample incubated in the water-hexadecane mixture relative to the sample incubated in water (OD_w+h_ / OD_w_ ×100).

### TEM and SEM imaging

For TEM and SEM imaging, a 1 mL sample with an OD_600_ of 20 was centrifuged at 10,000 *g* for 1 min, and the pellet was fixed overnight with a 3.13% (vol/vol) glutaraldehyde solution in 0.1 M PBS. After fixation, the samples were washed five times with PBS. Post-fixation was performed using a 1% osmium tetroxide solution in Milli-Q water for 1 h. To remove the excess of chemicals, samples were rinsed three times with Milli-Q water. Samples for TEM imaging were stained with uranyl acetate (0.5%, wt/vol) in Milli-Q water for 10 min. For both TEM and SEM imaging, samples were then dehydrated through a series of ethanol-in-water solutions of increasing concentration (vol/vol): 30% for 5 min, 50% for 5 min, 70% for 5 min, 96% for 5 min, and 100% ethanol twice for 10 min each. TEM samples underwent two additional 10 min dehydration steps with acetone, followed by progressive replacement with an acetone/epoxy resin mixture. Afterward, the samples were immersed in pure resin, transferred to molds, and incubated at 70°C for 3 days. For TEM imaging, ultrathin sections of 70 nm were obtained, stained with Reynolds’ solution (34), and later visualized with the equipment JEOL JEM 1010 (Akishima, Tokio, Japón). On the other hand, the SEM specimens were subjected to critical point drying using a Leica EM CPD300 system (Wetzlar, Germany) after the ethanol washes. The dried samples were mounted on aluminum stubs with carbon adhesive tape, gold-/palladium-coated in a BAL-TEC SCD 005 vacuum evaporator (Pfäffikon, Switzerland), and analyzed under a JEOL JSM 6360-LV microscope.

### Permeability assays

To evaluate coat permeability, a 4 kDa FITC-dextran probe was used as previously described (35). Briefly, spore samples at an OD_600_ of 10 were incubated at 4°C in the presence of 0.625 mM of the fluorescent probe. At several time points, spores were extracted, diluted 10-fold in distilled water, and pelleted by centrifugation at 13,500 *g* for 5 minutes. Subsequently, the pelleted spores were washed with distilled water twice. Samples were processed using a Nikon Eclipse E4000 microscope (Nippon Kogaku KK, Japan) equipped with an epifluorescence unit. In all cases, a × 100 objective was used with immersion oil, giving a total magnification of ×1,000. Images of fields containing 25–50 free spores were captured using *ZEN Blue* software (v 3.5.093.00002, Carl Zeiss Microscopy GmbH, Oberkochen, Germany). An exposure time of 3,000 ms was used for image acquisition, with excitation and emission filters at wavelengths of 470 nm and 520 nm, respectively. Measurements of over 100 spores per condition were processed identically from three biological replicates collected and assayed for fluorescence on different days. Finally, the position of the probe and fluorescence signal quantification was obtained using the Ellipsoid Localization Microscopy MATLAB analysis script (36), selecting the ellipsoidal model and a pixel width of 53.7 nm.

### Statistical analysis

One-way ANOVA with Tukey’s *post hoc*, or unpaired parametric *t*-test when comparing two conditions, was performed using GraphPad PRISM 5.0. Differences were considered statistically significant when *P* was ≤0.05. Data in the figures correspond to averages and standard deviations calculated from three biological replicates, which were obtained on different working days, unless otherwise indicated.

## RESULTS

### Nutrient-induced germination of spores produced at optimal and reduced a_w_

**Fig 1:**
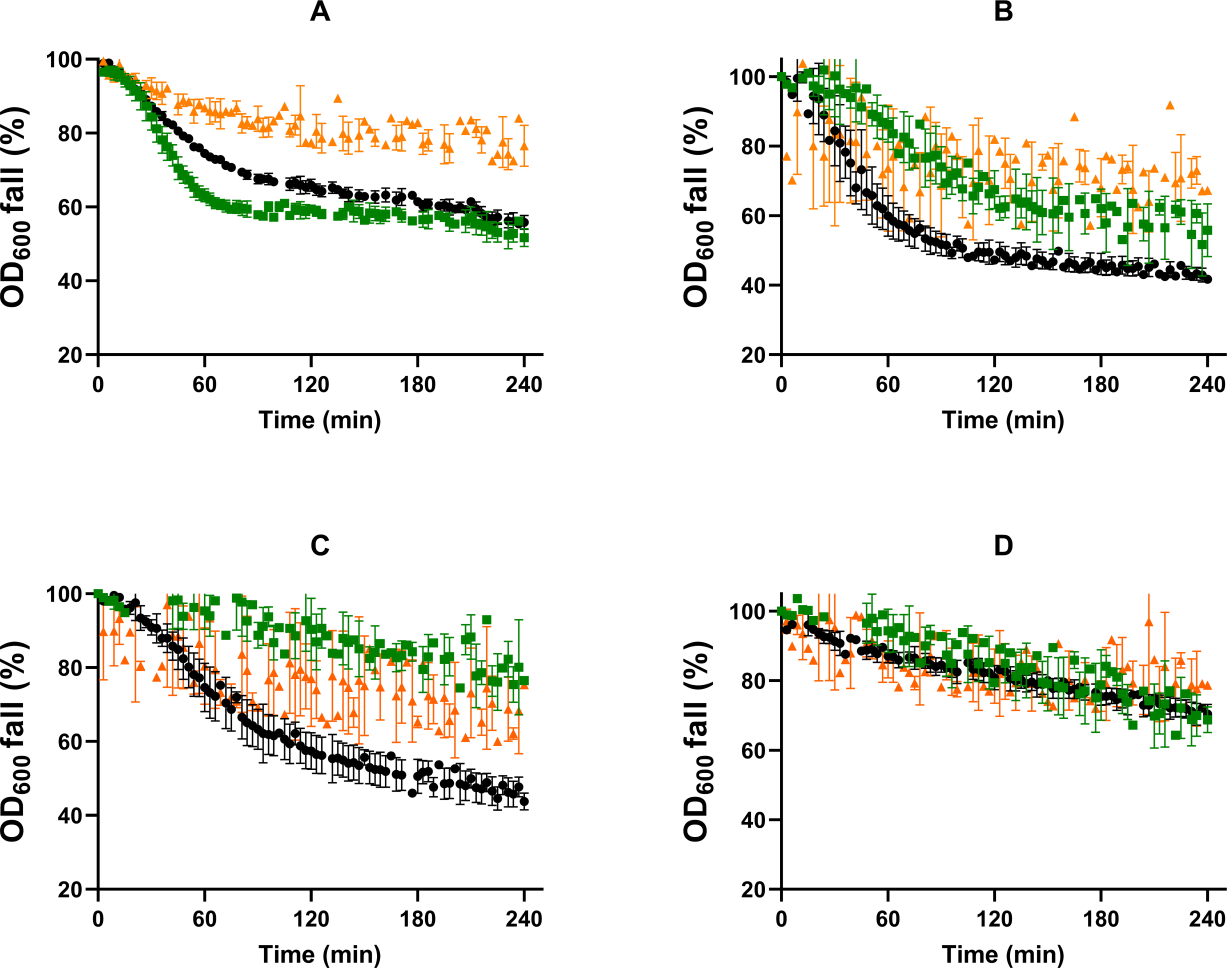
Germination curves obtained by spectrophotometry of *B. subtilis* spores produced under optimal (~ 0.99; ●, S_control_) and reduced *a_w_* (0.98) with different solutes (■, with NaCl, S_salt_; ▲, with glycerol, S_gly_) in (**A**) NBYE, (**B**) L-alanine, (**C**) L-valine, or (**D**) AGFK. Data in the figures correspond to averages and standard deviations calculated from three biological replicates.

**Fig 2:**
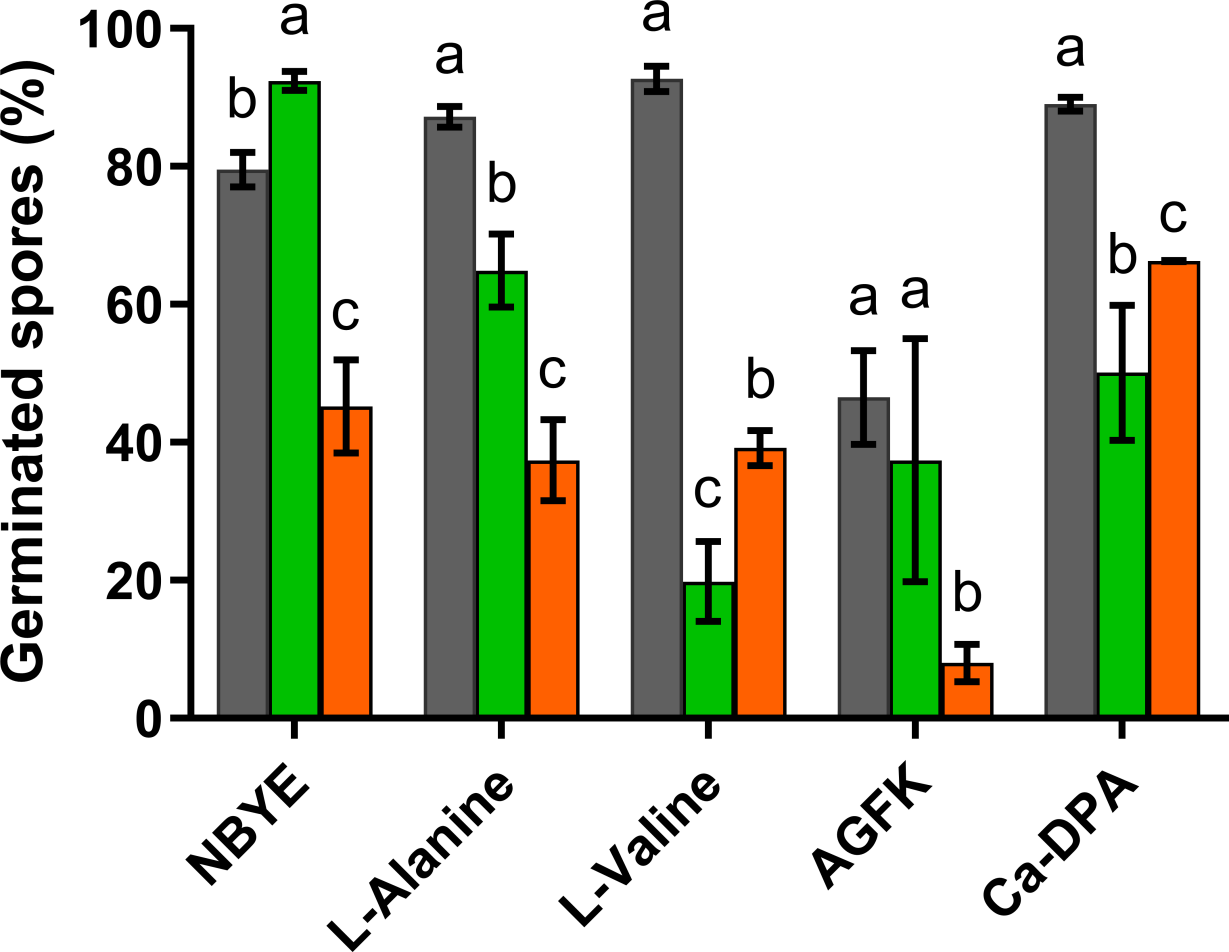
Germination efficiency after 4 h exposure to nutrients in S_control_ (■), S_salt_ (■), and S_gly_ (■) spores without prior heat activation. Data in the figures correspond to averages and standard deviations calculated from three biological replicates. Different lowercase letters indicate statistically significant differences (*P* ≤ 0.05) among spore populations produced at different *a_w_* conditions for each germinant.

Germination of *B. subtilis* spores produced at optimal (~ 0.99; S_control_) and reduced *a_w_* (0.98) adding different solutes (NaCl, S_salt_, or glycerol, S_gly_) was evaluated in a rich growth medium (NBYE) and in buffer enriched with L-alanine, L-valine, or AGFK. Germination kinetics were first examined by measuring the decrease in OD_600_ over time (Fig. 1). The percentages of germinated spores (both phase-dark and -gray spores) at the end of the 4 h assay are depicted in Fig. 2.

Spores formed at reduced *a_w_* behaved differently from control spores depending on the solute used to depress *a_w_* and the nutrient stimuli. In NBYE, S_salt_ spores exhibited more rapid germination over S_control_ populations, reaching 13% more germinated spores (*P* ≤ 0.05; Fig. 2). On the other hand, the germination efficiency of S_gly_ populations was around half of that observed in spores produced under optimal conditions (*P* ≤ 0.05; Fig. 2). In L-alanine and L-valine, the germination efficiency of S_salt_ spores was lower than that of S_control_ spores, with a greater decrease in the latter nutrient (*P* ≤ 0.05; Fig. 2). S_gly_ spores displayed a decrease in the germination extent in both amino acids, similar to that observed in NBYE (approximately 2.0-fold *P* ≤ 0.05; Fig. 2). In AGFK, reducing *a_w_* of the sporulation medium altered germination kinetics when glycerol, but not salt, was used as a depressor (Fig. 1). S_gly_ spores showed a 5.8-fold decrease in the germination efficiency compared to S_control_ spores (*P* ≤ 0.05; Fig. 2), which was much higher than that observed for the other nutrients. Interestingly, the spore counts determined by microscopy of both S_salt_ and S_gly_ populations matched that given by plate counts in NAYE after 24 h of incubation (*P* > 0.05; Fig. S1), indicating that most spores produced at reduced *a_w_* were indeed able to respond to nutrients, albeit at a much slower rate.

We noticed that S_gly_ spores were prone to aggregation, which may contribute to the large scatter observed in the OD_600_ curves (Fig. 1). In addition, a large heterogeneity in brightness loss was observed among germinated S_gly_ spores, with a large proportion of spores not achieving total loss of refractility (i.e., gray-phase spores), likely indicating partial retention of DPA. Therefore, to confirm that lowering the *a_w_* of the sporulation medium impaired the response of spores to nutrients, the germination kinetics of S_control_, S_salt_, and S_gly_ spores in the two single amino acids and in AGFK were assessed by DPA-Tb fluorometry (Fig. S2). No statistically significant differences were observed between the percentage of DPA release and the percentage of germinated spores determined microscopically at the end of the 4 h experiment (*P* > 0.05; Table S2). Thus, these data confirmed the lower germination efficiency of S_gly_ spores than S_control_ spores in the three nutrients and that S_salt_ spores showed impaired germination rate and efficiency only in L-alanine and more remarkably in L-valine (Table S2 and Fig. S2).

### Effect of heat activation on nutrient-induced germination of spores produced at optimal and reduced a_w_

**Fig 3:**
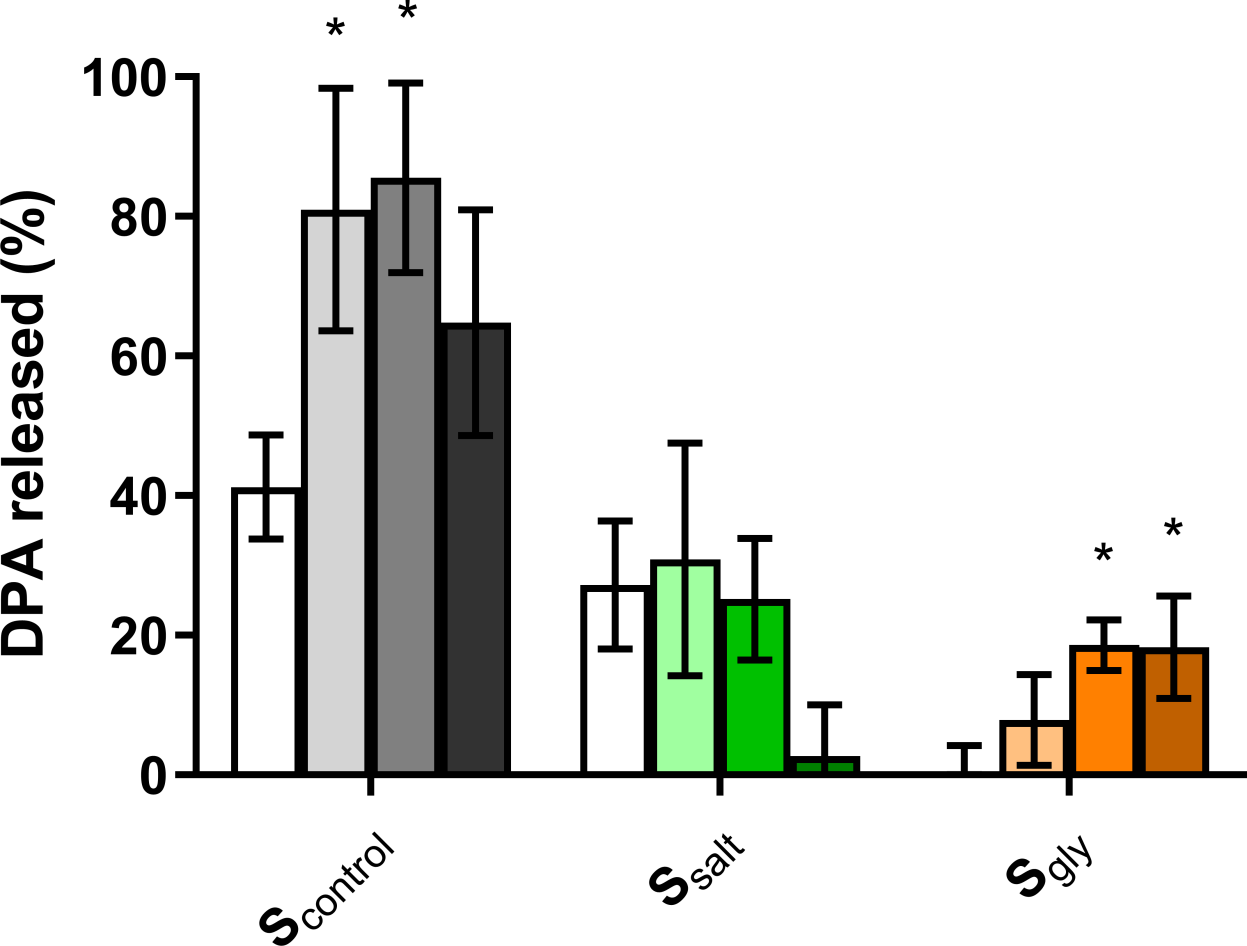
Percentage of DPA released after 4 h exposure to AGFK in S_control_, S_salt_, and S_gly_ spores without (white bars) and with prior heat activation (65°C, light color bars; 75°C, medium color bars; and 85°C, dark color bars). Data in the figures correspond to averages and standard deviations calculated from three biological replicates. Asterisks indicate statistically significant differences (*P* ≤ 0.05) between non-heat-activated and heat-activated spores within each population.

Given that some germination defects resulting from changes in sporulation temperature of *B. subtilis* spores can be alleviated by heat activation (17, 37), we tested whether heat treatment at various intensities (65°C, 75°C, or 85°C for 30 min) could improve germination of spores produced at reduced *a_w_* in L-alanine and AGFK. L-valine results were excluded for simplicity as heat activation did not improve germination of the control populations. To circumvent potential issues by aggregation and heterogeneity of spores, these experiments were performed using DPA-Tb fluorometry. Figure 3 shows the difference in the percentage of DPA released at the beginning and at the end of the 4 h germination experiment as heat activation induced leakage of DPA to varying degrees depending on the treatment intensity and sporulation condition (Fig. S3 and S4).

Treatment at 65°C or 75°C did not affect the percentage of DPA released at 4 h in L-alanine in S_control_ and S_gly_ spores (*P* > 0.05; Fig. S3). However, the 75°C treatment reduced the fraction of DPA released in L-alanine in S_salt_ spores by 2.7-fold, along with the rate of fluorescence increase (*P* ≤ 0.05; Fig. S3), thereby exacerbating the differences in germination kinetics between S_control_ and S_salt_ in this nutrient. As previously reported for this strain (17), AGFK-induced germination was the nutrient stimulus that benefited the most from heat activation in control spores (Fig. S3 and S4). The DPA released by S_control_ spores increased approximately 2.0-fold when exposed to 65°C or 75°C for 30 min and to a lesser extent (1.6-fold) when activated at 85°C (*P* ≤ 0.05; Fig. 3). In contrast, none of the heat treatments significantly changed the germination of S_salt_ populations compared to non-activated spores (*P* > 0.05; Fig. 3). Therefore, although no differences in germination kinetics were observed between non-heat-activated S_control_ and S_salt_ spores in AGFK (Fig. 1; Fig. S4), spores produced at high salinity did show impaired germination rate and efficiency when spores were heat-activated at mild temperatures (Fig. 3; Fig. S4). S_gly_ spores showed increased DPA release after exposure to the most intense treatments (75°C and 85°C), but neither sample reached the same level as S_control_ spores activated at the same temperature (Fig. 3).

### Chemical-induced germination of spores produced at optimal and reduced *a*_*w*_

**Fig 4:**
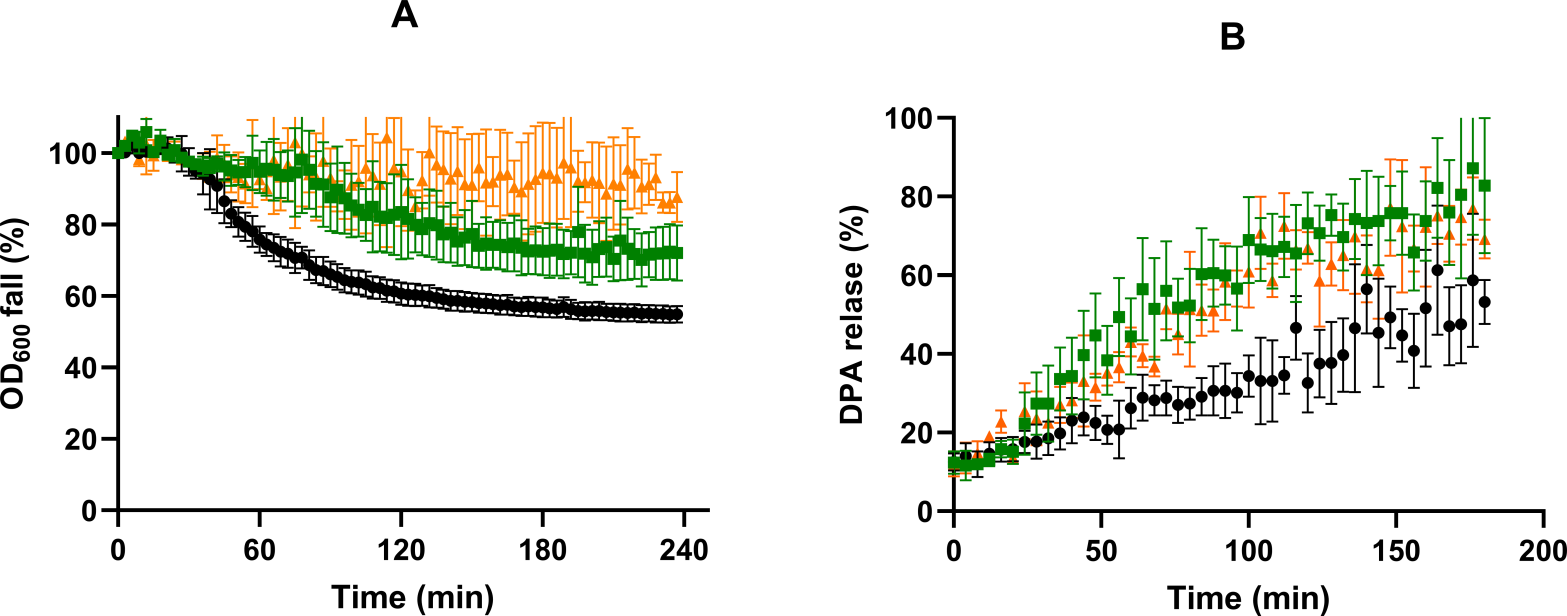
Germination curves of S_control_ (●), S_salt_ (■), and S_gly_ (▲) spores in (**A**) Ca-DPA and (**B**) dodecylamine. Germination in Ca-DPA represents the percentage of OD_600_ decrease, while germination in dodecylamine corresponds to the percentage of DPA released from the total population content. Data in the figures correspond to averages and standard deviations calculated from three biological replicates.

We also examined whether spores produced at reduced *a_w_* exhibited defects in GR-independent germination induced by Ca-DPA or dodecylamine (Fig. 4). Ca-DPA initiates germination by activating the cortex lytic enzyme CwlJ located in the inner coat layer (38), whereas dodecylamine induces DPA release likely through activation of SpoVAC channels (39, 40). Ca-DPA germination curves of S_salt_ spores showed increased *delay* phase and decreased rate of OD_600_ fall compared to S_control_ spores (Fig. 4A), and consequently, a 1.8-fold decrease in the germination efficiency (*P* ≤ 0.05; Fig. 2). The percentage of OD_600_ decrease in response to Ca-DPA was lower for spores produced in the presence of glycerol (< 15%) than with salt (Fig. 4A). However, the proportion of germinated S_gly_ spores determined microscopically was higher than in S_salt_ populations (*P* ≤ 0.05; Fig. 2). This may be attributed to the observation that the proportion of gray-phase spores (counted as germinated spores) was greater in S_gly_ than in S_salt_ populations, and higher than in nutrient-germinated S_gly_ samples. In contrast to the Ca-DPA stimulus, the rate of DPA release in response to dodecylamine was faster in both S_salt_ and S_gly_ spores than in S_control_ spores (Fig. 4B). For instance, control spores released 30% of their total DPA after 100 min exposure to dodecylamine, whereas spores obtained at reduced *a_w_*, released about 70% at the same time, regardless of the solute used.

### Changes in the coat caused by sporulation at increased salinity conditions are associated with germination defects of S_salt_ spores

**Fig 5:**
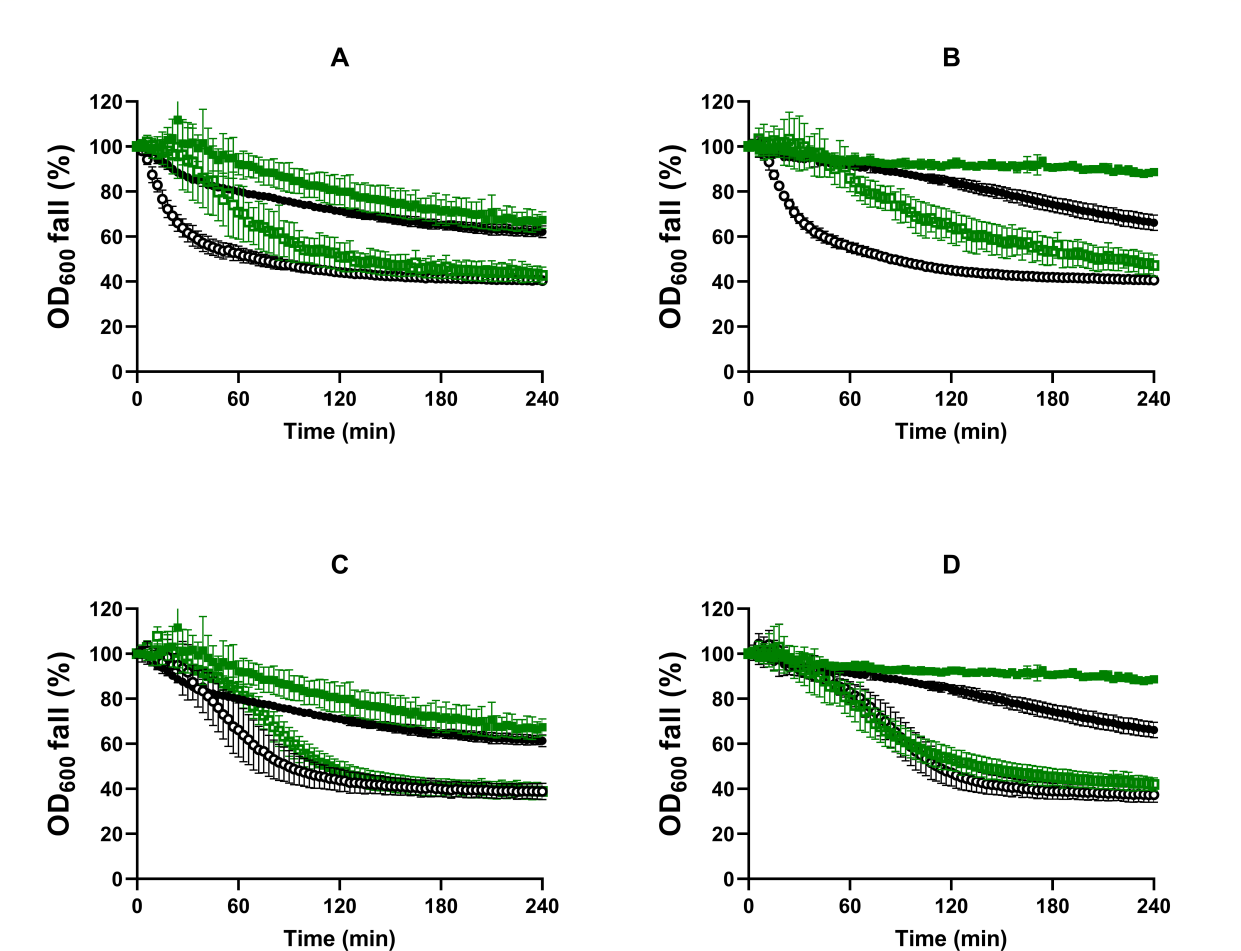
Germination curves obtained by spectrophotometry of WT (solid symbols), Δ*cotY* (A, B; open symbols), and Δ*cotE* (C, D; open symbols) spores produced under optimal (black symbols: ● – S_control_ WT; ○ – S_control_ Δ*cotY*/*cotE*) or reduced *a_w_* with NaCl (green symbols: ■ – S_salt_ WT; □ – S_salt_ Δ*cotY*/*cotE*) in L-alanine (**A, B**) or L-valine (**C, D**). Data in the figures correspond to averages and standard deviations calculated from three biological replicates.

We hypothesized that impaired nutrient germination of spores produced at reduced *a_w_* may involve alterations in coat properties because their behavior coincided with others previously reported in coat-altered spores: (i) the ineffectiveness of heat activation in enhancing nutrient germination (Fig. 3) (25, 35), (ii) the impaired germination in Ca-DPA (Fig. 4A) (25, 35); and (iii) the increased rate of DPA release in the presence of dodecylamine (Fig. 4B) (25). To test this hypothesis, we compared the germination of mutant spores with morphogenesis defects in different coat layers with wild-type (WT) spores. The mutants chosen were Δ*cotY*, in which the crust is disrupted (41–43), and Δ*cotE*, which lacks both the crust and the outer coat (43, 44). The study focused on spores produced at depressed *a_w_* (0.98) with NaCl as increased salinity during sporulation is a far more feasible scenario in both industrial and natural environments than elevated glycerol media. The germination was only assessed by spectrophotometry due to the inhibitory effect of Tb^3+^ on the germination of Δ*cotE* spores (45, 46). Germination was induced with the nutrients L-alanine, L-valine, and AGFK, without prior heat activation unless otherwise mentioned, and with the GR-independent agent Ca-DPA (Fig. 5; Fig. S5 and S7).

CotY- and CotE-deficient spores produced at either optimal or reduced *a_w_* germinated in L-alanine and L-valine at a higher rate and to a greater extent than WT spores (*P* ≤ 0.05; Fig. 5; Table S3). However, the germination curves of Δ*cotE* spores showed a delay phase. The germination efficiency of Δ*cotY* and Δ*cotE* spores in L-alanine and L-valine did not change (*P* > 0.05) when sporulated in the presence of salt, but only S_salt_ Δ*cotY* spores still exhibited a slower germination rate than that of S_control_ Δ*cotY* spores, especially in L-valine (*P* ≤ 0.05; Fig. 5; Table S3).

In AGFK, the Δ*cotY* spores germinated to a similar extent as the WT spores independently of the *a_w_* of the sporulation medium (*P* > 0.05; Table 1). However, *k* values were significantly higher in S_control_ Δ*cotY* spores than in S_control_ WT, and in turn higher than in S_salt_ Δ*cotY* populations (*P* ≤ 0.05; Table 1; Fig. S5). The absence of CotE significantly increased the germination rate and efficiency compared to Δ*cotY* and WT spores (*P* ≤ 0.05), with no noticeable differences between populations produced at different *a_w_* (*P* > 0.05; Table 1; Fig. S5). Since heat activation enhanced the response of WT spores to AGFK produced at optimal but not at reduced *a_w_* with NaCl (Fig. 3), Δ*cotE* spores were also subjected to a heat activation treatment (55°C, 30 min). While this heat activation improved germination of S_control_ WT spores, but not S_salt_ WT spores, it could significantly increase both the germination rate and efficiency of Δ*cotE* spores regardless of the sporulation condition (*P* ≤ 0.05; Table 1; Fig. S6).

**TABLE 1:** Germination rate and the percentage of germination at the end of the assay (4 h) of non-heat-activated and heat-activated (55°C, 30 min) WT, Δ*cotY,* and Δ*cotE* spores, produced under optimal (S_control_) or reduced *a_w_* with NaCl (S_salt_), in AGFK^*a*^

Germinant	Population	*k* (min^−1^)	R^2^	RMSE	Efficiency (%)
AGFK (non-heat-activated)	S_control_ WT	0.0069^a^ (0.0019)	0.969	0.401	42.1^a^ (6.4)
	S_salt_ WT	0.0056^a^ (0.0034)	0.941	1.151	46.4^a^ (5.9)
	S_control_ Δ*cotY*	0.0132^b^ (0.001)	0.994	0.616	52.9^a^ (6.3)
	S_salt_ Δ*cotY*	0.0084^a^ (0.0019)	0.991	0.824	51.5^a^ (3.7)
	S_control_ Δ*cotE*	0.0220^c^ (0.0036)	0.924	3.400	75.4^bc^ (4.53)
	S_salt_ Δ*cotE*	0.0247^c^ (0.0010)	0.998	0.466	69.4^b^ (2.11)
AGFK (heat-activated)	S_control_ WT	0.0180^a*^ (0.0037)	0.998	0.532	77.0^a*^ (3.3)
	S_salt_ WT	0.0055^b^ (0.0018)	0.960	1.671	43.1^b^ (12.6)
	S_control_ Δ*cotY*	N.D.	N.D.	N.D.	N.D.
	S_salt_ Δ*cotY*	N.D.	N.D.	N.D.	N.D.
	S_control_ Δ*cotE*	0.0455^c*^ (0.0087)	0.995	0.848	83.4^a*^ (1.3)
	S_salt_ Δ*cotE*	0.0395^c*^ (0.0076)	0.996	0.670	90.7^a*^ (6.2)

^*a*^ The values in brackets correspond to the standard deviations of the means calculated from three biological replicates. Different lowercase letters indicate statistically significant differences (*P* ≤ 0.05) among strains and sporulation conditions for each germinant and heat treatment condition. An asterisk indicates statistically significant differences (*P* ≤ 0.05) between non-heat-activated and heat-activated spores within each strain and sporulation condition. N.D.: not determined. The Δ*cotY* spores were not heat-activated because the germination rate of S_control_ Δ*cotY* and S_salt_ Δ*cotY* populations was significantly (*P* ≤ 0.05) different.

Ca-DPA was able to induce germination in Δ*cotY* spores, but not in Δ*cotE* spores (Fig. S7) likely due to the loss of CwlJ (47, 48), which in nutrient germination could be compensated by other enzymes present in Δ*cotE,* spores such as SleB (47, 49). The Δ*cotY* populations produced under optimal conditions showed significantly higher *k* values but lower proportions of germinated cells than S_control_ WT spores (Table 2; Fig. S7). While both germination rate and germination efficiency were impaired when WT spores were sporulated with a high salt concentration, S_control_ and S_salt_ Δ*cotY* spores showed identical values, which in turn were equal to those of S_salt_ WT populations (*P* > 0.05; Table 2).

The fact that the absence of CotY alleviated the negative effects of sporulation at increased salinity on germination efficiency, but not completely on germination rate, in L-alanine and L-valine, and that the absence of CotE restored both germination efficiency and rate in both amino acids, as well as the response to heat activation in AGFK, suggests that alterations in the coat may be involved in the impaired response of S_salt_ WT spores.

**TABLE 2:** Germination rate and the percentage of germination at the end of the assay (4 h) of WT, Δ*cotY*, and Δ*cotE* spores, produced under optimal (S_control_) or reduced *a_w_* with NaCl (S_salt_), in Ca-DPA^*a*^

Germinant	Population	*k* (min^−1^)	R^2^	RMSE	Efficiency (%)
Ca-DPA	S_control_ WT	0.0132^a^ (0.0012)	0.999	0.337	84.8^a^ (6.9)
	S_salt_ WT	0.0077^b^ (0.0035)	0.923	0.964	40.1^b^ (7.8)
	S_control_ Δ*cotY*	0.0218^c^ (0.0022)	0.975	1.285	57.4^c^ (1.1)
	S_salt_ Δ*cotY*	0.0138^ab^ (0.0060)	0.981	1.159	46.9^b^ (7.6)
	S_control_ Δ*cotE*	N.M.	N.M.	N.M.	4.0^d^ (1.8)
	S_salt_ Δ*cotE*	N.M.	N.M.	N.M.	2.3^d^ (2.1)

^*a*^ The values in brackets correspond to the standard deviations of the means calculated from three biological replicates. Different lowercase letters indicate statistically significant differences (*P* ≤ 0.05) among strains and sporulation conditions for each germinant. N.M.: not modeled. OD_600_ values did not decrease more than 15%.

### Hydrophobicity of spores produced at optimal conditions and increased salinity

Since the defect in nutrient- and Ca-DPA-induced germination caused by sporulation at high salinity was attenuated in the Δ*cotY* mutant and CotY is known to play a role in the spore surface properties (50), we aimed to determine whether the coat modifications in S_salt_ spores could lead to differential hydrophobicity. The hydrophobicity of the spore surface plays an important role in the interaction with germinants and consequently in the different response to each agent with different hydrophobicity, such as L-alanine and L-valine (51). Therefore, the BATH assay was performed on WT and mutant spores produced under optimal and reduced *a_w_* (0.98) adding NaCl (Fig. S8). As expected (50), spores from the Δ*cotY* mutant, as well as S_control_ Δ*cotE* populations, were more hydrophobic than WT spores. However, no statistically significant differences (*P* > 0.05) in hydrophobicity were found between spores cultivated at optimal and reduced *a_w_* in the WT or coat morphogenesis-deficient mutants, suggesting that sporulation at high salinity did not alter germination by changing the hydrophobicity of the spore coat.

### TEM and SEM imaging of spores produced under optimal conditions and increased salinity

**Fig 6:**
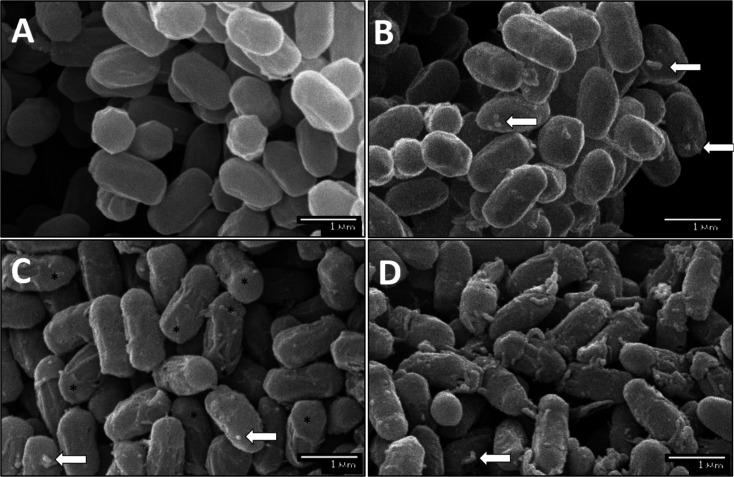
Representative SEM images of (**A, B**) WT and (**C, D**) Δ*cotY* spores produced at (**A, C**) optimal (~ 0.99) and (**B, C**) reduced *a_w_* (0.98) with NaCl. Samples were processed in the same batch and on the same day, as described in Materials and Methods. White arrows indicate the deformations mentioned in the text, while “*” shows the “cap-like” material remaining in Δ*cotY* spores.

To assess structural changes in the coat layers induced by sporulation at high salinity, S_control_ and S_salt_ WT spores were imaged by TEM (Fig. S9). Although the crust was imperceptible because its visualization requires staining with ruthenium red (52), we could distinguish the inner coat and the outer coat. No significant differences were observed in the outer coat or inner coat thickness between sporulation conditions (*P* > 0.05; data not shown). SEM observation of the WT and Δ*cotY* spores produced under optimal and high salinity conditions was also performed to assess the state of the crust. As shown in Fig. 6, the outermost layer of S_control_ WT spores exhibited a smooth surface, while S_control_ Δ*cotY* spores had a striated structure in the equatorial region, corresponding to the outer coat (53), and residual material at the poles. These observations agree with the results by Bartels et al. (41). No statistically significant differences in size were observed between S_salt_ and S_control_ spores of the WT strain (*P* < 0.05; data not shown). However, S_salt_ WT spores exhibited irregular formations on the surface, in contrast to the uniformity observed in S_control_ WT spores. Irregular formations, similar to those observed in S_salt_ WT spores, were present in S_control_ Δ*cotY* populations, and their presence was accentuated in the latter strain. Our results differ from those of previous imaging of *B. subtilis* ATCC 31324 spores produced at *a_w_* 0.950 depressed with NaCl, which showed a smoother surface and smaller size than spores produced under optimal conditions (19, 54). Such discrepancy could be due to differences between strains and/or the composition of the sporulation medium.

### FITC permeation in spores produced under optimal conditions and increased salinity

**Fig 7:**
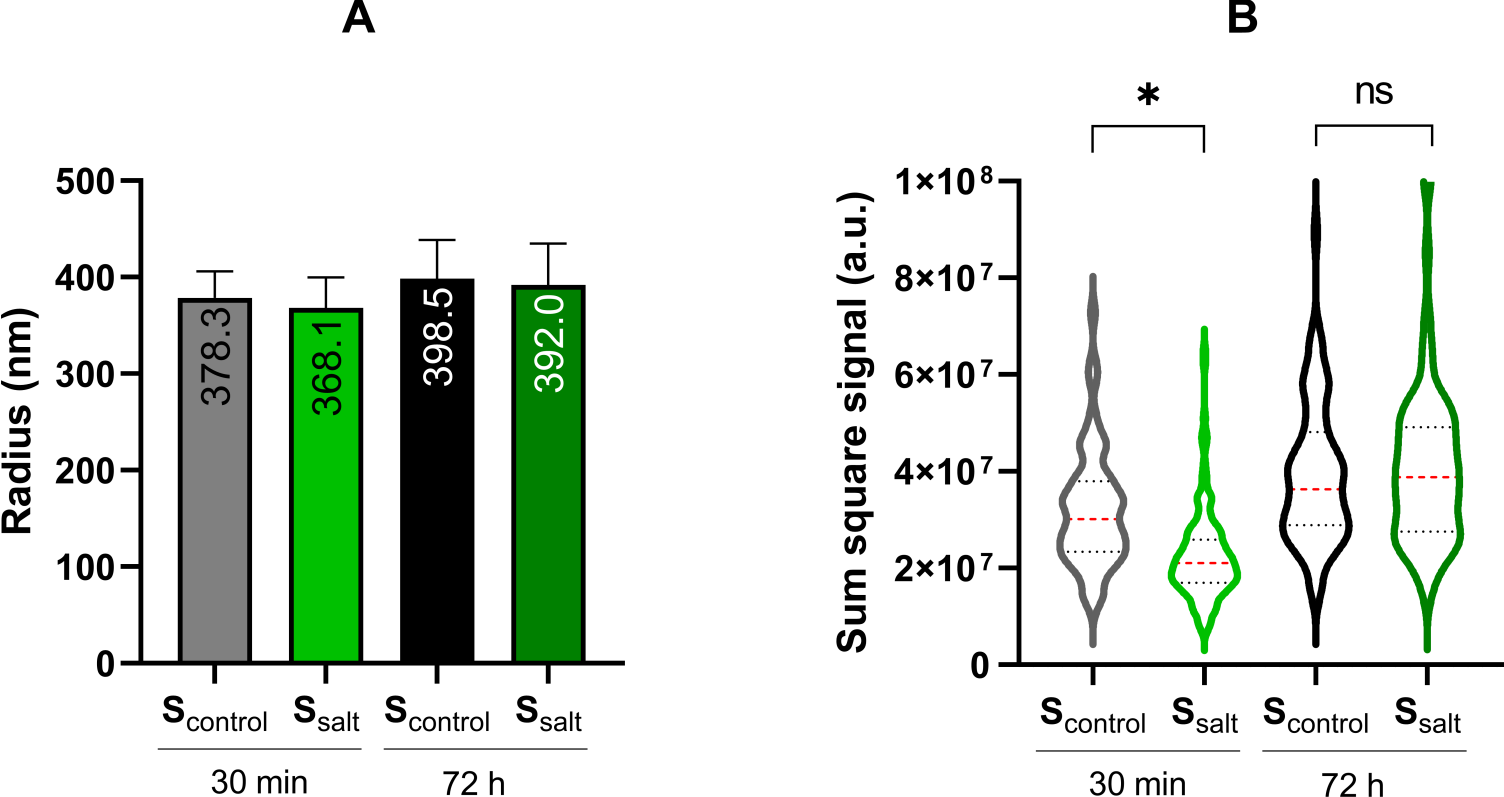
Permeation of 4 kDa FITC-dextran in individual S_control_ and S_salt_ WT spores at different incubation times. (**A**) Position of the probe from the center, with the numbers indicating the mean value expressed in nanometer. (**B**) Sum of pixel intensities for each spore after subtraction of uniform background noise. Data represent measurements of over 100 individual spores, processed identically from three biological replicates collected and assayed for fluorescence on different days. The red discontinuous line indicates the mean value of the population, while the upper and lower gray discontinuous lines indicate the 75th and 25th percentiles, respectively. The asterisk indicates statistically significant differences (*P* ≤ 0.05) between S_control_ and S_salt_ populations at each time point.

To assess whether coat alterations induced by sporulation at increased salinity led to permeability changes, the kinetics of 4 kDa FITC-dextran probe uptake in WT spores produced under optimal and high salinity conditions were tested. This probe was previously reported to permeate *B. subtilis* spores to a depth equivalent to that of the outer coat (35). We observed that after only 30 min of incubation, the probe was located around 380–370 nm from the center of the spore, regardless of the sporulation condition (Fig. 7A). This depth corresponds to the location of the outer coat in our spores, as confirmed by TEM imaging measurements (Fig. S9). The mean location of the probe increased slightly with incubation time (72 h, *P* > 0.05), suggesting that the dye accumulated in the spores (Fig. 7A).

When dye uptake in S_control_ and S_salt_ spores was quantified over time, both populations showed a significant increase in fluorescence after 30 min compared to unstained spores (*P* ≤ 0.05, data not shown). The increase in the mean total fluorescence intensity was around half in S_salt_ spores compared to S_control_ spores (*P* ≤ 0.05 Fig. 7B). Conversely, after 72 h of incubation, no significant differences were observed between the two populations. These outcomes demonstrated slower probe uptake in S_salt_ spores than in S_control_ spores, possibly due to reduced outermost coat layer permeability.

## DISCUSSION

The effect of sporulation temperature is widely recognized as an important factor contributing to variability in spore germination. However, the impact of *a_w_* has been overlooked so far, despite the fact that environments with lower humidity than ideal culture media, such as soil, are common sporulation niches (1). This research demonstrates that *B. subtilis* populations sporulated at depressed *a_w_* (0.98) by the addition of either NaCl (S_salt_ spores) or glycerol (S_gly_ spores) exhibited impaired germination compared to spores incubated under optimal conditions (*a_w_* ~0.99, S_control_ spores). The degree of this impairment varied depending on the solute and the type of germinant. We showed, both by quantifying the proportion of phase-dark cells and/or DPA release, that spores produced in the presence of glycerol exhibited a lower germination efficiency than S_control_ spores in NBYE, L-alanine, L-valine, and especially in AGFK. Furthermore, S_gly_ spores displayed a slower rate of DPA release in the three specific nutrients. Similarly, spores produced at high salinity showed both a lower germination rate and efficiency in L-alanine and especially in L-valine, in which the extent of germinated spores in S_salt_ populations was lower than in S_gly_ spores. Time-lapse microscopy will be necessary to assess intrapopulation heterogeneity and identify the germination steps that are compromised. The fact that populations produced at the same *a_w_* with salt or glycerol behave differently suggests that each solute, in addition to increasing osmotic stress, alters the germination capabilities of spores through specific mechanisms. Glycerol readily diffuses passively across the cell membrane, whereas NaCl has a lower permeability. In addition, each solute could differently alter the protein profiles of spores cultured at the same *a_w_* (54). Further studies will be needed to determine exactly how each solute influences spore properties beyond the depression of *a_w_*.

Variations in germination kinetics induced by changes in sporulation conditions have been attributed to modifications in the levels of GR, properties of the inner membrane, and/or the coat (18, 55, 56). Differential expression of GR has been linked to the variations in germination kinetics induced by changes in sporulation temperature and nutrient richness (55). Although we do not know if changes in the levels of GerA, GerB, and/or GerK occurred in S_salt_ spores, our results suggest that a general reduction in GR levels could not fully explain their impaired nutrient germination phenotype. This is because S_salt_ spores showed better germination fitness in NBYE than S_control_ spores, and all three GRs are known to be essential for efficient germination in complex nutrient media (28). In addition, the degree of germination impairment on S_salt_ spores differed between L-alanine and L-valine, even though both act on GerA (57). Conversely, downregulation of GR expression may be consistent with the phenotypes of S_gly_ spores, in which the proportion of superdormant spores, i.e., spores that respond slowly or are unable to respond (13), in all the nutrients exceeded 50%, and those able to germinate displayed a slower rate of DPA release in L-alanine, L-valine, and AGFK. However, heat activation did not improve the germination of S_gly_ spores to the same extent as S_control_ spores at any of the temperatures tested (65 °C–85 °C). This contrasts with the behavior of nutrient-superdormant spores, which are known to contain lower levels of GR compared to their dormant counterparts (13, 58) and still respond to heat activation, albeit at higher temperatures, than the whole population from which they originate (59).

Both S_salt_ and S_gly_ populations also exhibited decreased germination rate and efficiency when germination was induced by bypassing GR activation by Ca-DPA. Several non-exclusive reasons could contribute to explain this phenomenon: i) variations in CwlJ expression (26), ii) differences in the cortex structure affecting the hydrolytic activity of the aforementioned enzymes (60), iii) alterations in coat structure leading to attenuated CwlJ activity, and/or iv) lower permeability to exogenous Ca-DPA (25). The first three explanations would be consistent with the defective germination of S_gly_ spores in all agents tested, as loss of CwlJ would reduce the germination rate of these spores by impeding Ca-DPA-induced germination or by delaying cortex hydrolysis and core hydration in nutrient-induced germination (31, 61, 62). However, a defect in CwlJ function, either through decreased expression, reduced hydrolytic activity due to structural differences in the cortex, or altered coat environment, would not be able to explain the maintenance or even improvement in germination fitness observed in S_salt_ spores in AGFK and NBYE, respectively. Furthermore, the fact that ∆*cotY* spores germinated indistinguishably in Ca-DPA when sporulated at optimal and reduced *a_w_* with NaCl supports that S_salt_ WT spores do not undergo significant changes in the cortex structure or cortex hydrolytic enzymatic activity. Rather, this suggests a lower permeability to Ca-DPA for the differential response of S_salt_ spores compared to S_control_ spores in the WT strain.

Changes in the inner membrane properties have been proposed to contribute to the differences in response to heat activation and to dodecylamine (37, 63). Spores produced at suboptimal temperatures show greater improvement in nutrient germination by heat activation, particularly in AGFK, and a higher extent of dodecylamine germination than those formed at the optimal temperature, likely due to differences in inner membrane properties. Furthermore, *B. subtilis* strains with a larger number of copies of the *spoVA*^2mob^ operon have higher heat resistance and higher temperature requirements for heat activation, which has been attributed to the differential expression of proteins in the spore inner membrane likely affecting lipid mobility (64, 65). In addition, the presence of the *spoVA*^2mob^ operon negatively affects dodecylamine-induced germination (65, 66). The fact that heat activation, even at the highest temperature tested, did not stimulate the germination of S_salt_ spores and marginally increased the germination efficiency of S_gly_ spores in AGFK, despite both populations being more heat-resistant than S_control_ spores (24), together with the higher rate of DPA release in S_salt_ and S_gly_ populations than in S_control_ spores when exposed to dodecylamine, suggests that the germination defect of spores produced at reduced *a_w_* is not associated with changes in inner membrane properties.

The impaired germination of spores produced at reduced *a_w_* in nutrients and Ca-DPA, without much improvement after heat activation in the former stimulus, and their increased response to dodecylamine compared to S_control_ spores are consistent with the phenotypes of spores with altered coat permeability (25, 35). Indeed, by using coat-deficient mutants affecting different layers, Δ*cotY*, in which the crust is disrupted (41, 42), and Δ*cotE*, which lacks both the crust and the outer coat (43, 44), we could infer that alterations in the outermost coat layers may be involved in the germination defects of S_salt_ spores in nutrients and Ca-DPA. In addition, by comparing the rate of uptake of the 4 kDa FITC-dextran probe, we found that S_salt_ spores exhibited a decreased permeability to the dye, slowing its access to the outer coat. However, as spores lacking CotY, despite being one of the major structural proteins in this layer (41), exhibited a fragmented crust but not a complete absence, we cannot at this moment discern whether increased sporulation salinity only affects the crust or the outer coat components or both structures. Indeed, deletion of CotY reversed the decrease in germination efficiency but not in the germination rate in L-alanine and L-valine observed in S_salt_ WT spores. On the other hand, the germination kinetics in L-alanine, L-valine, and AGFK of Δ*cotE* spores were identical independently of the sporulation salinity. With respect to Ca-DPA, the germination efficiency of Δ*cotY* spores was lower than that of WT spores produced under optimal conditions and was maintained unchanged in S_salt_ Δ*cotY* spores, suggesting that at least the crust is relevant for Ca-DPA-induced germination. Although this study demonstrates that the crust and/or outer coat layer play a critical role in the impaired germination and unresponsiveness to heat activation of spores produced at increased salinity, changes in other spore structures, either independently or as a result of interactions with the coat, such as those known to occur between the inner membrane and this structure, may also contribute to the phenotype of spores produced at reduced *a_w_*.

The plausible changes experienced by the coat of spores obtained at high salinity that led to germination defects remain unknown. Isticato et al. (18) attributed the impaired germination in a mixture of L-asparagine or L-alanine, glucose, fructose, and potassium of *B. subtilis* PY79 spores produced at 42°C compared to those incubated at 37°C to changes in the outer coat. More specifically, spores prepared at the highest temperature exhibited a granular, less compact, and thicker outer coat than spores produced at 37°C, which coincided with a lower proportion of the morphogenetic protein CotH and its related proteins in the former, likely due to the low thermal stability of CotH. On the other hand, Abhyankar et al. (67) suggested that a higher degree of cross-linking in the crust and outer coat proteins in 8-day mature spores compared to 2-day mature spores of *B. subtilis* PY79 could be related to the more heterogeneous and on-average slower germination of the former. Perhaps, the assembly of the crust and outer coat and/or their amount of protein cross-linking is affected by sporulation at high salinity in *B. subtilis* spores, potentially influencing inner membrane mobility. Another explanation for the phenotypes of S_salt_ spores would be alterations in coat components that regulate permeability, such as the *gerP* operon, which encodes proteins thought to facilitate germinant access to GRs (25, 68, 69). It has been demonstrated that *B. subtilis* spores lacking the *gerP* operon exhibited impaired germination in nutrients, even after heat activation, and in Ca-DPA without affecting the colony-forming ability beyond 20% (25). Additionally, the deletion mutant showed increased sensitivity to dodecylamine (25). GerP is likely located in the inner coat, and its absence affects the assembly of the upper coat layers (35), particularly the crust, which is apparently absent (31), and hampers the penetration of germinants (25, 35, 68). The nutrient germination defect in spores of *B. subtilis*, *B. anthracis*, and *B. cereus* with abnormal GerP production was suppressed by either deletion of the *cotE* gene or chemical decoating (25, 68, 69), which is in agreement with the fact that sporulation at a high salt concentration did not affect the germination of mutant spores lacking the crust and outer coat (i.e., Δ*cotE*). In this sense, alterations in other coat components thought to be involved in efficient nutrient accessibility to GR, such as GerY, remain a possibility (70). Further analyses, such as coat proteomics, are needed to better understand the changes suffered by spores produced at increased salinity.

Even more intriguing is the selectivity of sporulation at high salinity for slowing down the germination in different types of nutrients, with the negative effect being greater for L-valine, followed by L-alanine and AGFK. The higher suppression of germination in L-valine than in L-alanine in S_salt_ spores compared to S_control_ spores in the WT strain could be explained by the different hydrophobicity of the two amino acids, with L-valine possessing an extra methyl group. However, no difference in spore hydrophobicity was found between sporulation at optimal and increased salinity, so other factors should be further investigated to elucidate these discrepancies. On the other hand, coat alterations induced by sporulation at reduced *a_w_* did not seem to alter permeability to small molecules such as TbCl_3_. This molecule is known to seriously compromise germination in coat-defective spores (45, 46), while we did not observe discrepancies between germination curves obtained by spectrophotometry and DPA-Tb fluorometry in S_salt_ WT spores. Further research is needed to elucidate the mechanisms of the selective permeability of coat layers in intact spores to further understand the functional changes undergone by the coat components in S_salt_ spores in the WT and in the coat-deficient mutants.

In conclusion, this work provides the first evidence that lowering *a_w_* of the sporulation medium with either glycerol or NaCl results in *B. subtilis* spores with impaired germination in most nutrients, which defect could not be reversed by thermal activation, and in Ca-DPA. In the case of spores formed under high salinity, we could infer that changes in the crust and/or outer coat, resulting in decreased permeability, may be involved in their germination defect. Environments with lower humidity than ideal culture media, such as soil, are frequent sporulation niches (1), and soil salinization is expected to increase with climate change (20, 21). Therefore, the knowledge gained from this study, together with the influence of other sporulation factors and inherent interspecific and intraspecific variations, provides valuable data to assess the variability in the behavior of spores contaminating environments and foods. Furthermore, contaminating spores originated from environments with reduced *a_w_*, which exhibited elevated heat resistance compared to those produced under optimal conditions (24), may also pose a substantial challenge in the design of effective germination-inactivation approaches due to their slower response to germination stimuli.

## SUPPLEMENTAL MATERIAL

Supplemental material aem.00677-25-s0001.docx Tables S1 to S3; Fig. S1 to S9.

ASM does not own the copyrights to Supplemental Material that may be linked to, or accessed through, an article. The authors have granted ASM a non-exclusive, world-wide license to publish the Supplemental Material files. Please contact the corresponding author directly for reuse.
