# Supplementary material for: Sporulation at reduced water activity impairs germination kinetics of Bacillus subtilis spores
Source: Appl Environ Microbiol. 2025 Jun 10;91(7):e00677-25. doi: 10.1128/aem.00677-25 (PMC12285231; doi:10.1128/aem.00677-25)
Supplement: Supplemental material — Tables S1 to S3; Fig. S1 to S9. [file aem.00677-25-s0001.docx]

**Supplementary Information**

**Sporulation at Reduced Water Activity Impairs Germination Kinetics of *Bacillus subtilis* Spores**

Víctor Freire, Santiago Condón, Elisa Gayán*

**Table S1.** Primers used in this investigation.

| **Primer name** | **Sequence (5’→3’)** |
| --- | --- |
| *cotY*_Fw | TACAGCAACAAATACACTCG |
| *cotY*_Re | CAGTCTTCTTCAACAGCG |
| *cotE*_Fw | GCACACACCATTTCCAACCA |
| *cotE*_Re | TACAGTTCCGGGCGTAATCA |

**Table S2.** Percentage of germinated spores and DPA released after 4-h exposure of S_control_, S_salt_, and S_gly_ populations to different nutrients. The values in brackets correspond to the standard deviations of the means calculated from three biological replicates. Different lowercase letters indicate statistically significant differences (*P* ≤ 0.05) among spore populations produced at different *a_w_* conditions for each germinant. Different capital letters indicate statistically significant differences (*P* ≤ 0.05) among germinants within each population sporulated at a specific condition.

| **Germinant** | **Population** | **Germination**  **efficiency (%)** | **DPA released (%)** |
| --- | --- | --- | --- |
| NBYE | S_control_ | 79.5^bA^ (2.5) | N.D. |
|  | S_salt_ | 92.4^aA^ (1.4) | N.D. |
|  | S_gly_ | 45.2^cA^ (11.7) | N.D. |
| L-alanine | S_control_ | 87.2^aB^ (1.5) | 96.71^aA^ (3.00) |
|  | S_salt_ | 64.9^bB^ (5.3) | 65.61^bA^ (6.68) |
|  | S_gly_ | 37.4^cA^ (10.2) | 33.10^cA^ (7.84) |
| L-valine | S_control_ | 92.7^aB^ (1.8) | 90.28^aA^ (3.64) |
|  | S_salt_ | 19.8^cC^ (5.8) | 12.46^cB^ (3.46) |
|  | S_gly_ | 39.2^bA^ (4.4) | 36.20^bA^ (12.71) |
| AGFK | S_control_ | 46.5^aC^ (6.8) | 44.80^aB^ (5.37) |
|  | S_salt_ | 37.4^aC^ (17.6) | 35.21^abC^ (4.00) |
|  | S_gly_ | 8.0^bC^ (4.7) | 18.20^bA^ (14.13) |

**Table S3.** Germination kinetic parameters (*delay* phase and gemination rate – *k*) and the percentage of germination at the end of the assay (4 h) of WT, Δ*cotY*, and Δ*cotE* spores, produced under optimal (S_control_) or reduced *a_w_* with NaCl (S_salt_), in L-alanine and L-valine. The values in brackets correspond to the standard deviations of the means calculated from three biological replicates. Different lowercase letters indicate statistically significant differences (*P* ≤ 0.05) among strains and sporulation conditions for each germinant.

| **Germinant** | **Population** | ***delay* (min)** | ***k* (min^-1^)** | **R^2^** | **RMSE** | **Efficiency (%)** |
| --- | --- | --- | --- | --- | --- | --- |
| L-alanine | S_control_ WT | N.D. | 0.0102^a^ (0.0009) | 0.990 | 1.066 | 64.4^a^ (2.9) |
|  | S_salt_ WT | N.D. | 0.0051^b^ (0.0008) | 0.965 | 2.238 | 56.0^b^ (4.6) |
|  | S_control_ Δ*cotY* | N.D. | 0.0326^c^ (0.0068) | 0.988 | 1.483 | 97.9^c^ (1.6) |
|  | S_salt_ Δ*cotY* | N.D. | 0.0164^d^ (0.0037) | 0.966 | 3.130 | 97.1^c^ (1.5) |
|  | S_control_ Δ*cotE* | 31.1^a^ (11.5) | 0.0284^cd^ (0.0043) | 0.991 | 1.995 | 95.5^c^ (3.4) |
|  | S_salt_ Δ*cotE* | 44.3^a^ (8.9) | 0.0212^cd^ (0.003) | 0.984 | 2.973 | 95.8^c^ (1.7) |
| L-valine | S_control_ WT | N.D. | 0.0149^a^ (0.0013) | 0.899 | 1.392 | 58.4^a^ (3.8) |
|  | S_salt_ WT | N.D. | 0.0074^b^ (0.0017) | 0.920 | 0.902 | 17.2^b^ (8.1) |
|  | S_control_ Δ*cotY* | N.D. | 0.0288^c^ (0.0009) | 0.988 | 1.678 | 94.7^c^ (7.2) |
|  | S_salt_ Δ*cotY* | N.D. | 0.0057^b^ (0.0018) | 0.969 | 2.884 | 92.4^c^ (10.7) |
|  | S_control_ Δ*cotE* | 49.7^a^ (7.1) | 0.0230^d^ (0.0033) | 0.975 | 3.673 | 99.1^c^ (0.1) |
|  | S_salt_ Δ*cotE* | 38.1^b^ (7.9) | 0.0189^ad^ (0.0043) | 0.988 | 1.460 | 95.1^c^ (4.9) |
| N.D.: not determined. Germination curves did not display a significant *delay* phase. | | | | | | |

**Figure S1:** Plate counts (CFU/mL; white bars) and microscopic counts (spores/mL; gray bars) of spores in S_control_, S_salt_, and S_gly_ suspensions. Data in the figures correspond to averages and standard deviations calculated from three biological replicates.

**
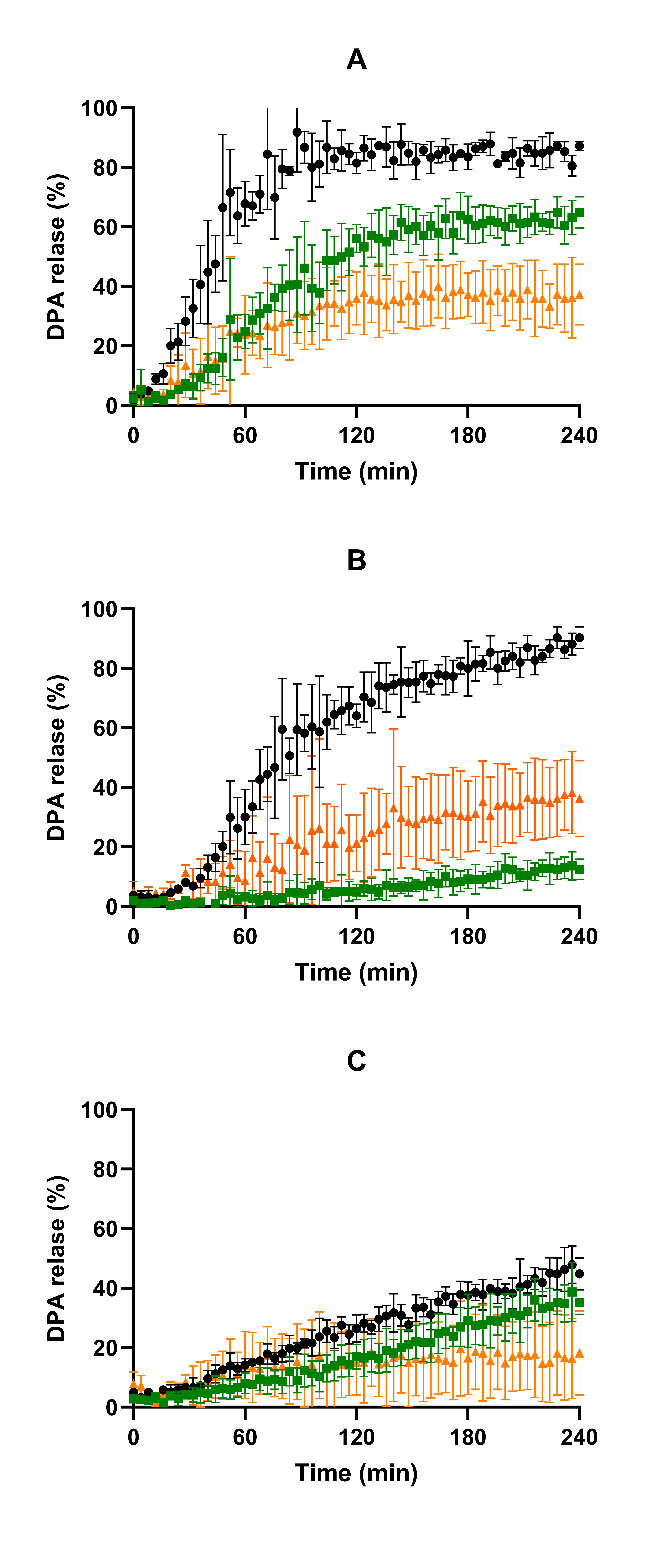
**

**Figure S2:** Germination curves obtained by DPA-Tb fluorometry of *B. subtilis* spores produced under optimal (~ 0.99; ●, S_control_) and reduced *a_w_* (0.98) with different solutes (■, with NaCl, S_salt_; ▲, with glycerol, S_gly_) in (A) L-alanine, (B) L-valine, or (C) AGFK. Data in the figures correspond to averages and standard deviations calculated from three biological replicates.


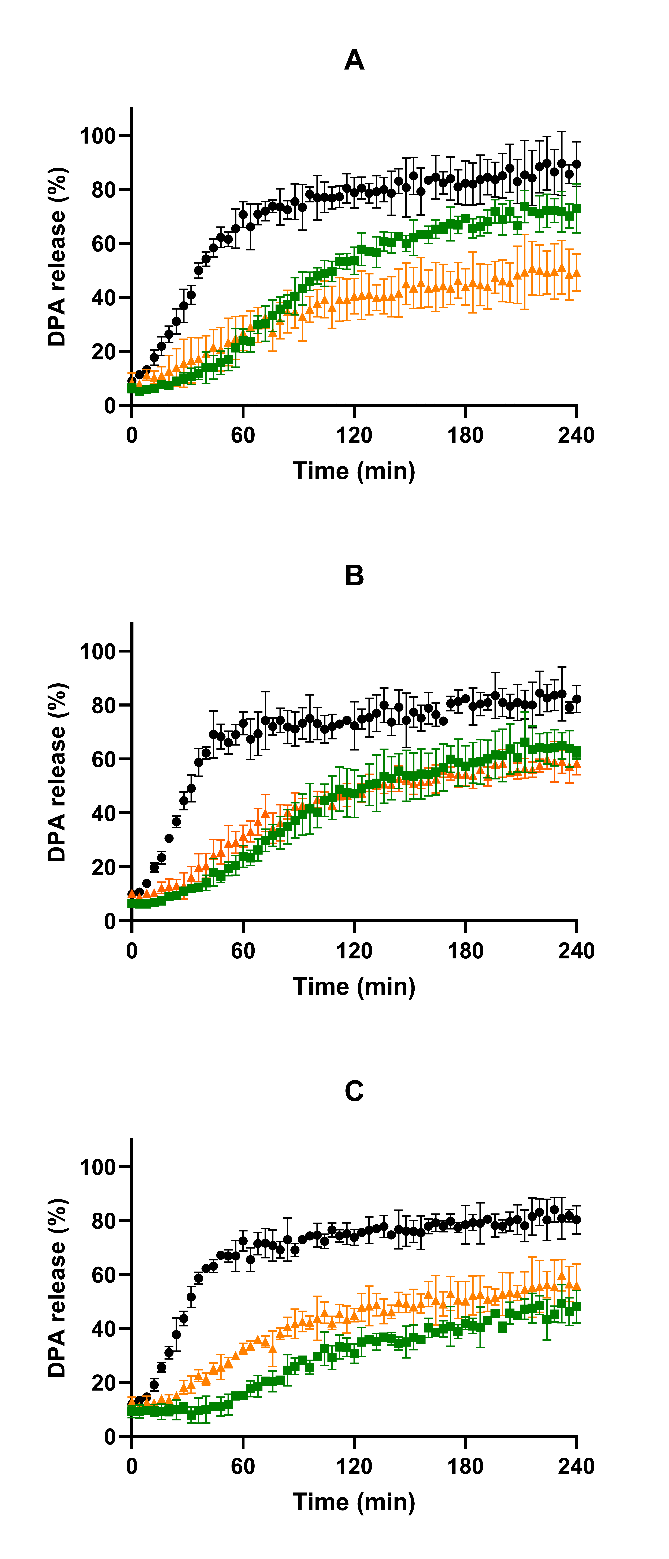


**Figure S3**: Germination curves obtained by DPA-Tb fluorometry of S_control_ (●), S_salt_ (■), and S_gly_ (▲) spores in L-alanine after exposure to different heat activation treatments: (A) no treatment, (B) 65 ºC for 30 min, or (C) 75 ºC for 30 min. Data in the figures correspond to averages and standard deviations calculated from four biological replicates.


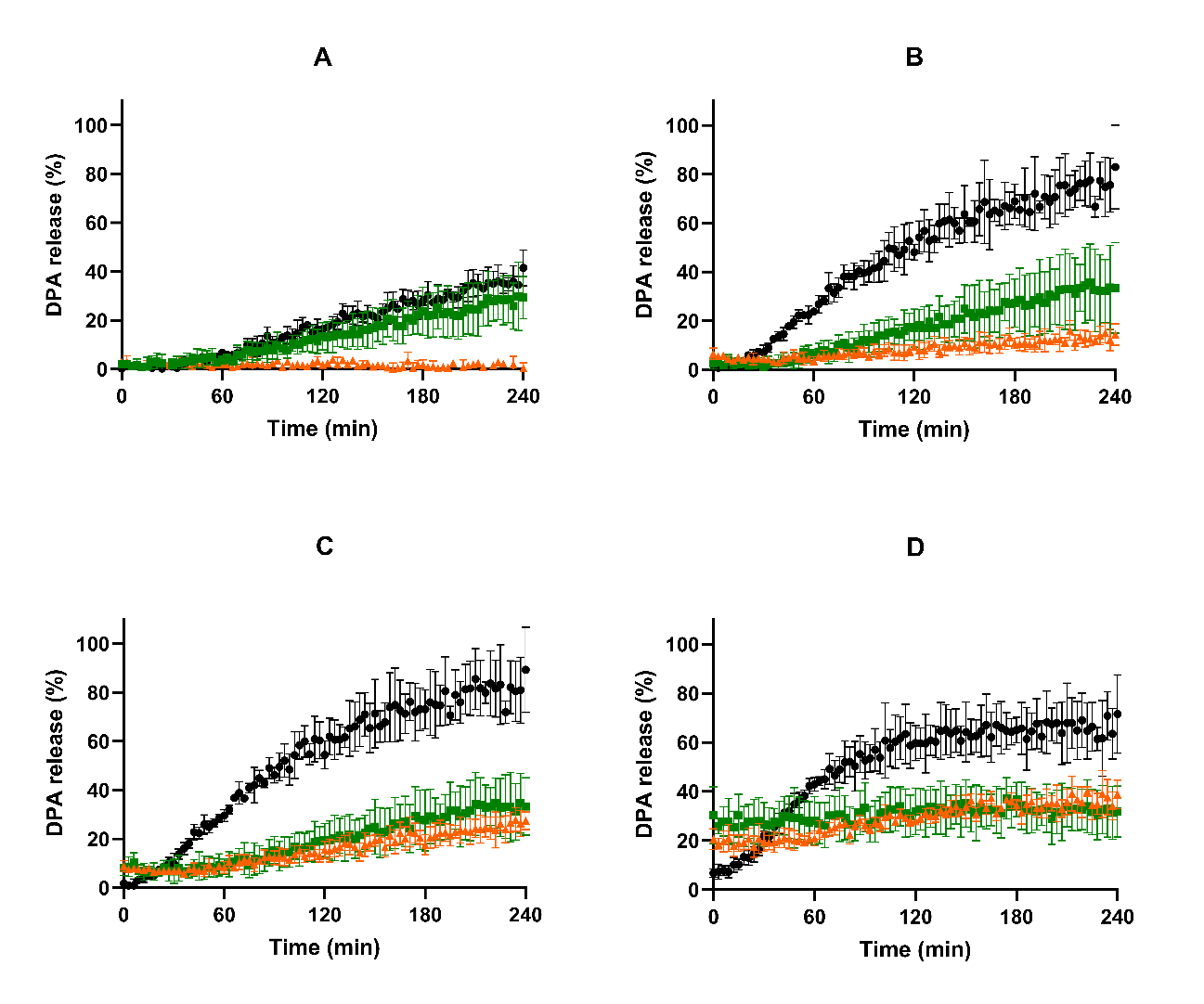


**Figure S4**: Germination curves obtained by DPA-Tb fluorometry of S_control_ (●), S_salt_ (■), and S_gly_ (▲) spores in AGFK after exposure to different heat activation treatments: (A) no treatment, (B) 65 ºC for 30 min, (C) 75 ºC for 30 min, or (D) 85 ºC for 30 min. Data in the figures correspond to averages and standard deviations calculated from four biological replicates.


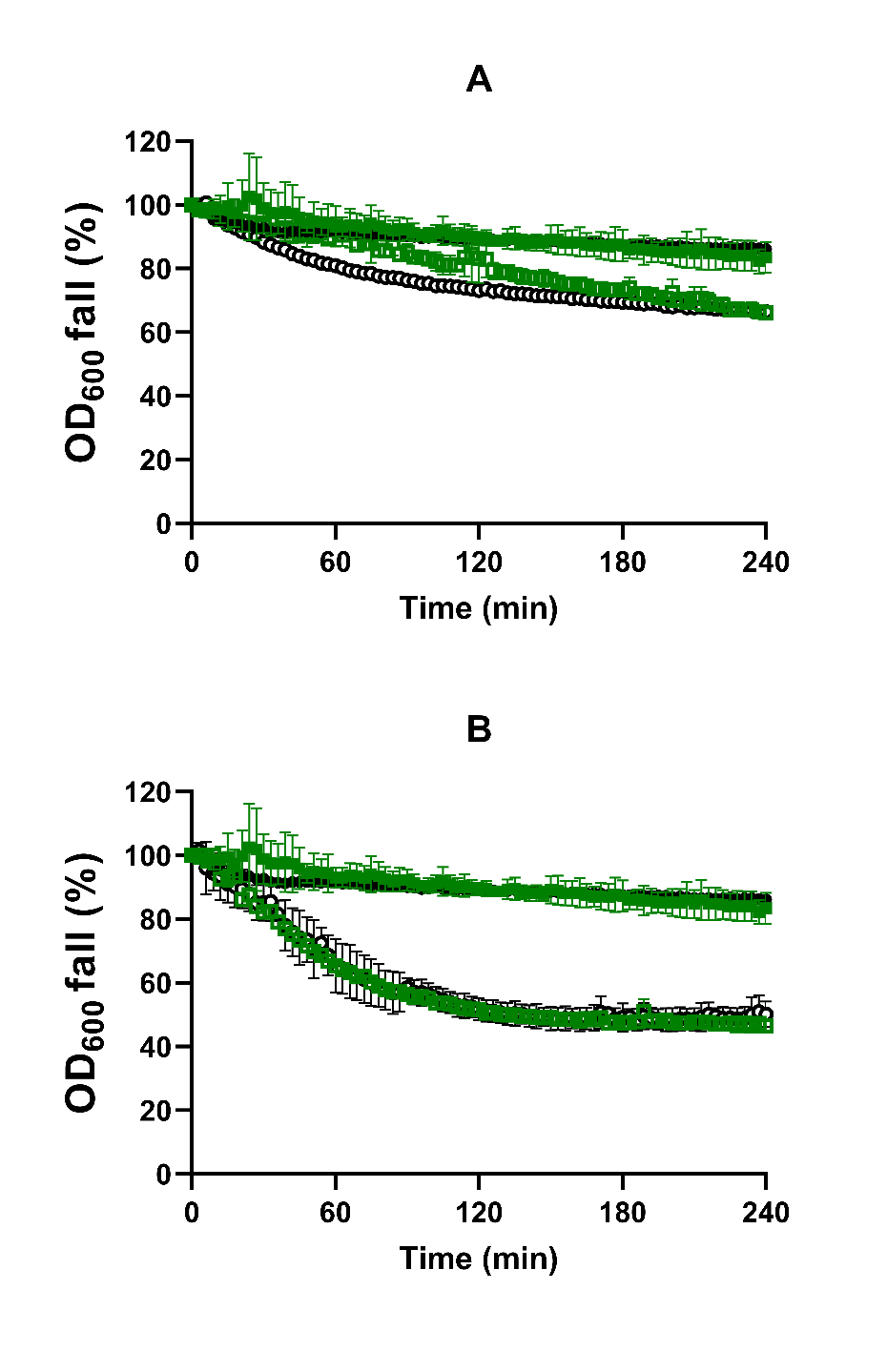


**Figure S5:** Germination curves obtained by spectrophotometry of WT (solid symbols), Δ*cotY* (A, open symbols), and Δ*cotE* (B, open symbols) spores produced under optimal (black symbols: ● – S_control_ WT, ○ – S_control_ Δ*cotY*/*cotE*) or reduced *a_w_* with NaCl (green symbols: ■ – S_salt_ WT, □ – S_salt_ Δ*cotY*/*cotE*) in AGFK. Data in the figures correspond to averages and standard deviations calculated from three biological replicates obtained on independent working days.


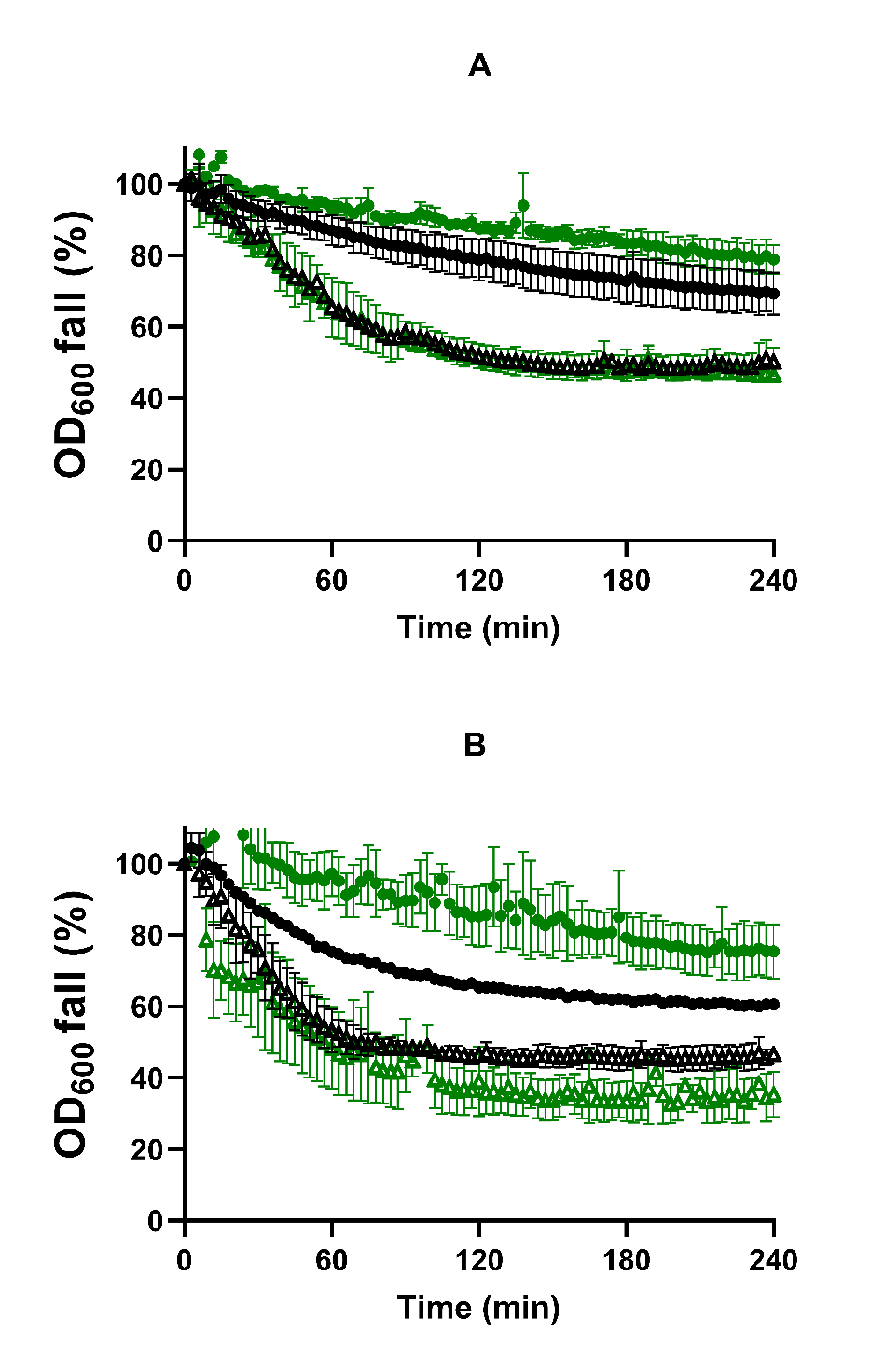


**Figure S6:** Germination curves obtained by spectrophotometry of (A) non-heat-activated and (B) heat activated (55 ºC, 30 min) WT (●, ●) and Δ*cotE* (∆, ∆) spores produced under optimal (black symbols: ● – S_control_ WT, ∆ – S_control_ Δ*cotE*) or reduced *a_w_* with NaCl (green symbols: ● – S_salt_ WT, ∆ – S_salt_ Δ*cotE*) in AGFK. Data in the figures correspond to averages and standard deviations calculated from three biological replicates.


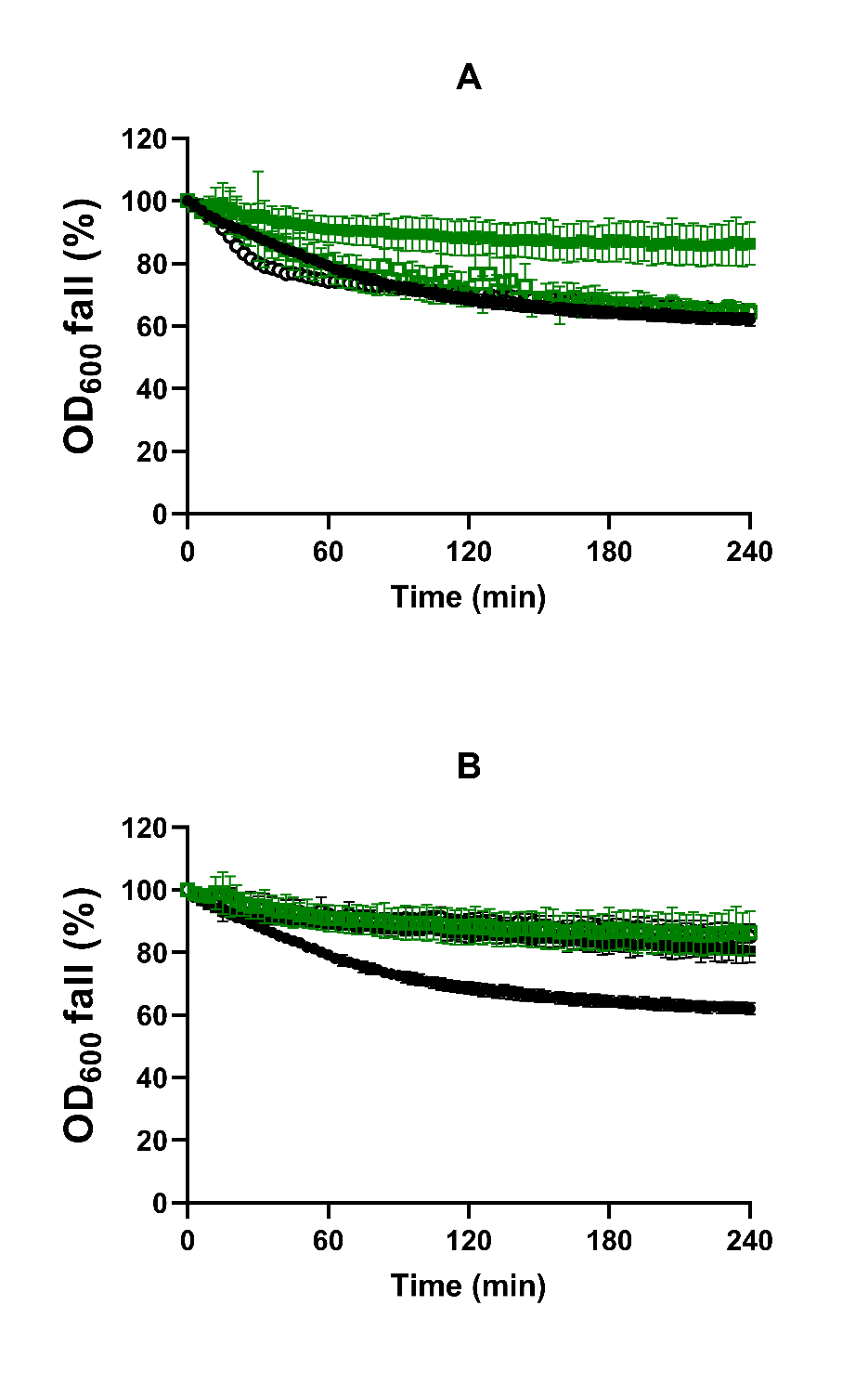


**Figure S7:** Germination curves obtained by spectrophotometry of WT (solid symbols), Δ*cotY* (A, open symbols), and Δ*cotE* (B, open symbols) spores produced under optimal (black symbols: ● – S_control_ WT, ○ – S_control_ Δ*cotY*/*cotE*) or reduced *a_w_* with NaCl (green symbols: ■ – S_salt_ WT, □ – S_salt_ Δ*cotY*/*cotE*) in Ca-DPA. Data in the figures correspond to averages and standard deviations calculated from three biological replicates.


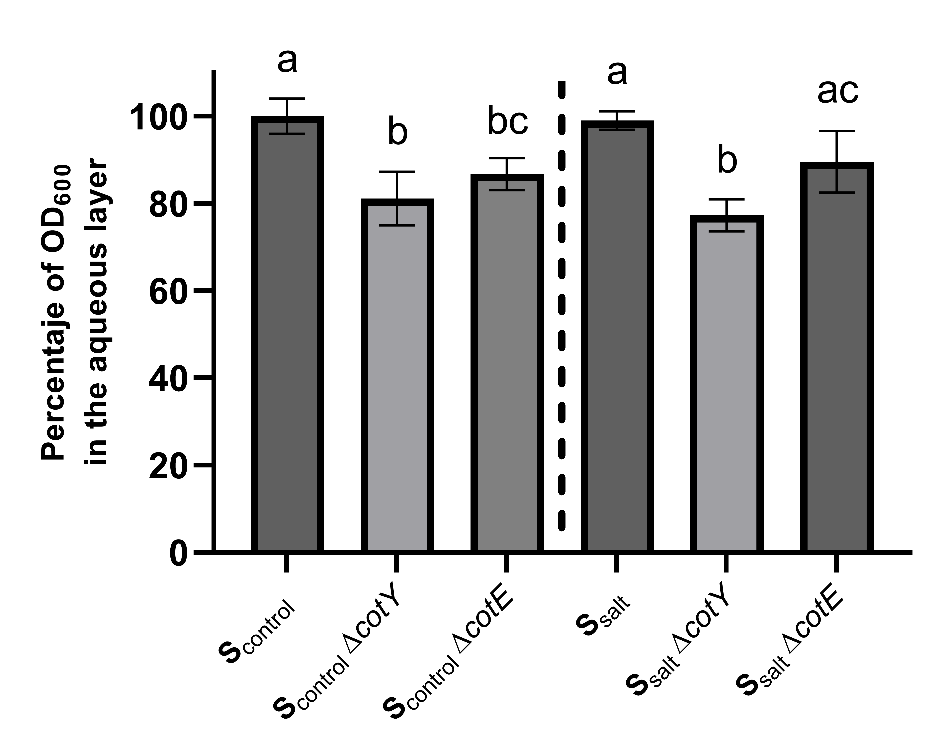


**Figure S8**. Surface hydrophobicity of WT and indicated coat morphogenesis-deficient spores, expressed as a percentage of the OD_600_ remaining in the aqueous phase using the BATH assay. Data correspond to averages and standard deviations calculated from two biological replicates. Different lowercase letters indicate statistically significant differences (*P* ≤ 0.05) among strains and sporulation conditions.

**
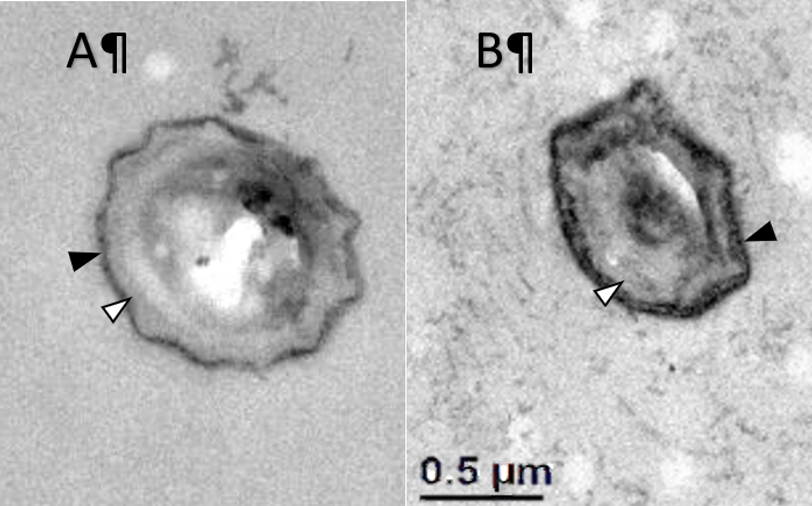
**

B

A

**Figure S9:** Representative TEM images of (A) S_control_ WT and (B) S_salt_ WT spores. Black arrows indicate the outer coat, while white arrows indicate the underlying inner coat. Samples were processed in the same batch and on the same day as described in the Experimental Procedures section.
